# Modeling the Potential Effects of New Tobacco Products and Policies: A Dynamic Population Model for Multiple Product Use and Harm

**DOI:** 10.1371/journal.pone.0121008

**Published:** 2015-03-27

**Authors:** Eric D. Vugrin, Brian L. Rostron, Stephen J. Verzi, Nancy S. Brodsky, Theresa J. Brown, Conrad J. Choiniere, Blair N. Coleman, Antonio Paredes, Benjamin J. Apelberg

**Affiliations:** 1 Resilience and Regulatory Effects, Sandia National Laboratories, Albuquerque, New Mexico, United States of America; 2 Center for Tobacco Products, U.S. Food and Drug Administration, Silver Springs, Maryland, United States of America; 3 Systems Research, Analysis and Applications, Sandia National Laboratories, Albuquerque, New Mexico, United States of America; 4 Policy and Decision Analytics, Sandia National Laboratories, Albuquerque, New Mexico, United States of America

## Abstract

**Background:**

Recent declines in US cigarette smoking prevalence have coincided with increases in use of other tobacco products. Multiple product tobacco models can help assess the population health impacts associated with use of a wide range of tobacco products.

**Methods and Findings:**

We present a multi-state, dynamical systems population structure model that can be used to assess the effects of tobacco product use behaviors on population health. The model incorporates transition behaviors, such as initiation, cessation, switching, and dual use, related to the use of multiple products. The model tracks product use prevalence and mortality attributable to tobacco use for the overall population and by sex and age group. The model can also be used to estimate differences in these outcomes between scenarios by varying input parameter values. We demonstrate model capabilities by projecting future cigarette smoking prevalence and smoking-attributable mortality and then simulating the effects of introduction of a hypothetical new lower-risk tobacco product under a variety of assumptions about product use. Sensitivity analyses were conducted to examine the range of population impacts that could occur due to differences in input values for product use and risk. We demonstrate that potential benefits from cigarette smokers switching to the lower-risk product can be offset over time through increased initiation of this product. Model results show that population health benefits are particularly sensitive to product risks and initiation, switching, and dual use behaviors.

**Conclusion:**

Our model incorporates the variety of tobacco use behaviors and risks that occur with multiple products. As such, it can evaluate the population health impacts associated with the introduction of new tobacco products or policies that may result in product switching or dual use. Further model development will include refinement of data inputs for non-cigarette tobacco products and inclusion of health outcomes such as morbidity and disability.

## Introduction

Despite decades of public health information about the health risks of cigarette smoking, smoking remains the cause of an estimated 480,000 premature deaths in the US each year [1] and is the leading cause of preventable disease and death throughout the world [2,3]. In the US, recent declines in cigarette smoking prevalence have coincided with an increase in the use of other traditional and non-traditional tobacco products. For example, total consumption of cigars more than doubled in the US from 2000 to 2011, rising from 6.2 billion to 13.7 over this time period [4]. The highest prevalence of cigar use occurs among youth and young adults [5,6], and cigars are now the most commonly used tobacco product among non-Hispanic black middle and high school students [6]. More recently, use of electronic cigarettes (e-cigarettes) has increased in popularity. Among youth, ever and current use of e-cigarettes doubled from 2011 to 2012 [6], following a similar pattern in adults from 2010 to 2011 [7]. In addition, polytobacco use is becoming increasingly common among tobacco users, particularly among young people. In 2012, more than 60% of middle and high school tobacco users reported using more than one type of tobacco product over the previous 30 days [8].

In the context of this diverse marketplace of tobacco products, the US Food and Drug Administration (FDA) has broad regulatory authority over the manufacture, marketing, and distribution of tobacco products. The FDA has the authority to permit the marketing of new tobacco products, to permit the marketing of modified risk tobacco products, which are tobacco products sold or distributed for use to reduce harm or the risk of tobacco-related disease associated with commercially marketed tobacco products, to establish product standards, and to educate the public about the risks of tobacco product use. In making regulatory decisions, such as those related to the marketing of tobacco products or the establishment of product standards, FDA must assess the risks and benefits of a particular regulatory action to the population as a whole, including users and non-users of tobacco products, while considering the impact of the action on the “increased or decreased likelihood that existing users of tobacco products will stop using such products” and “the increased or decreased likelihood that those who do not use tobacco products will start using such products” [9].

Statistical and computational modeling can be used to assess the potential impacts of tobacco regulatory actions. These tools have been used previously to analyze the effect of tobacco policy decisions on the public health by representing and forecasting cigarette use and harm in the US [10]. They can simulate status quo or baseline scenarios to project current or expected cigarette use and consequent health outcomes, as well as alternative or counterfactual scenarios to estimate differences in cigarette use patterns and health effects that may result from policy actions.

As an example, the SimSmoke model has been developed to predict the effects of tobacco control policies on population health outcomes. It uses a discrete time Markov process to simulate future smoking behavior in terms of initiation, cessation, and relapse and population change through births and deaths. SimSmoke has been used to model smoking prevalence and smoking-attributable deaths for the US from 2000 to 2040 under different intervention scenarios [11] and to model the effect of tobacco control policies on smoking outcomes in various countries, including, for example, Albania [12], Argentina [13], Brazil [14], Europe [15], Finland [16], Germany [17], Ireland [18], Italy [19], the Netherlands [20], South Korea [21], Sweden [22], Taiwan [23], Thailand [24] United Kingdom [25] and Vietnam [26].

A systems dynamics modeling approach has also been developed for predicting smoking prevalence [27]. This model uses age-specific initiation and cessation rates as well as birth and death rates to project smoking prevalence over time [28]. The model has been extensively used to project smoking prevalence for the US [29–31] and adapted for use in projecting smoking prevalence for 60 countries [32].

The Cancer Intervention and Surveillance Modeling Network (CISNET) lung cancer group is a consortium of researchers sponsored by the National Cancer Institute that investigates the association between smoking and lung cancer using a variety of statistical models and techniques. The group has developed detailed smoking histories for US birth cohorts from analysis of cross-sectional National Health Interview Survey (NHIS) data [33], analyzed trends in these cohort smoking histories [34], and modeled the impact of tobacco policies and smoking behavior on lung cancer and all-cause mortality in the US [35,36].

Other models have been developed to represent the population health effects of various health risk factors such as smoking for a variety of countries. For example, POHEM (the POpulation HEalth Model) [37], a discrete event microsimulation model developed by Statistics Canada, simulates the effect of multiple health risk factors such as smoking, obesity, and high blood pressure as well as socioeconomic factors such as educational attainment, marital status, and family income on disease incidence, progression, and mortality [38]. POHEM’s outcome measures include disease cases, deaths, disability-adjusted life expectancy, health care utilization, and health care expenditures for the population overall and for sub-populations. The model has generally been used with Canadian data.

The RIVM-CDM (Rijksinstitut voor Volksgezonheid en Milieu-Chronic Disease Model) is a discrete time deterministic simulation model [39]. The model simulates the effect of risk factors such as smoking and obesity on chronic disease incidence, prevalence, and mortality among members of a population cohort. The RIVM-CDM has been used to estimate the population health effects of behaviors such as smoking cessation [40] and the cost effectiveness of public health efforts such as youth smoking prevention programs [41]. The model has been designed specifically for use with data from the Netherlands.

More recently, DYNAMO-HIA (DYNAmic MOdeling for Health Impact Assessment) has been developed as a discrete time health risk microsimulation model [42]. The model simulates the risk factor history of individuals including smoking behavior and combines this information with simulated disease and mortality probabilities. The model has been used to estimate the population health effects of various risk factors including the effect of differences in smoking initiation and cessation rates on smoking prevalence, disease prevalence, and mortality in baseline and tobacco control policy scenarios [43]. DYNAMO-HIA has been designed for use with data from various European Union (EU) nations.

These models, although very useful, have typically focused on cigarette use in a population. Modeling efforts that have included two types of tobacco products have been much more limited. One such study employed Monte Carlo simulation of a decision tree model of tobacco initiation and use that included cigarette and snus use [44]. The model simulation results indicated that decreased cigarette use and increased snus use would not lead to reduction in population harm from tobacco products. Another study of cigarette and snus use employed a dynamic population model to estimate all-cause mortality for a hypothetical cohort based on input values for tobacco product initiation, cessation, and harm using Markov Chain Monte Carlo techniques. The model produced cohort life tables that are comparable to US and Swedish period life tables [45].

Given the wide variety of tobacco products on the market, the increasing prevalence of polytobacco use, and the need to assess the population health impact of new tobacco products and policies, this article presents the development of a multiple product, dynamical systems model. This model builds on previous work by incorporating multiple tobacco products and including a range of possible tobacco use behaviors, including product switching and dual use. In the present study, we demonstrate the use of this model by (1) projecting cigarette smoking prevalence and attributable mortality for the US population overall and by sex and age group and (2) simulating the introduction of a hypothetical new tobacco product with a range of values for product use and harm. In doing so, we demonstrate how the model can be used to examine the relative impacts of different assumptions about product use and risk and to identify those factors that most influence population health.

## Methods

The model simulates the effects of product initiation, switching, dual use, and cessation on future tobacco use and mortality in a population. The current formulation of the model can be used to compare the effects of various potential scenarios on future population health, as measured by all-cause mortality. In this analysis, we model a status quo scenario associated with use of cigarettes only and then compare this scenario to a range of scenarios characterized by the introduction of a hypothetical new tobacco product that presents lower mortality risk than cigarettes.

### Conceptual Model

The model tracks the size and characteristics of a population as well as tobacco use and harm within that population. The model is initiated with a starting population, divided into subgroups defined by age, sex, and tobacco use status (never, former, and current use) for each tobacco product included in the model. The model updates the population at each time step through births, net international migration, and deaths. The model also updates the size of population subgroups at each time step based on changes in tobacco product use and mortality by means of a first order, discrete Markov process.

**Fig 1:**
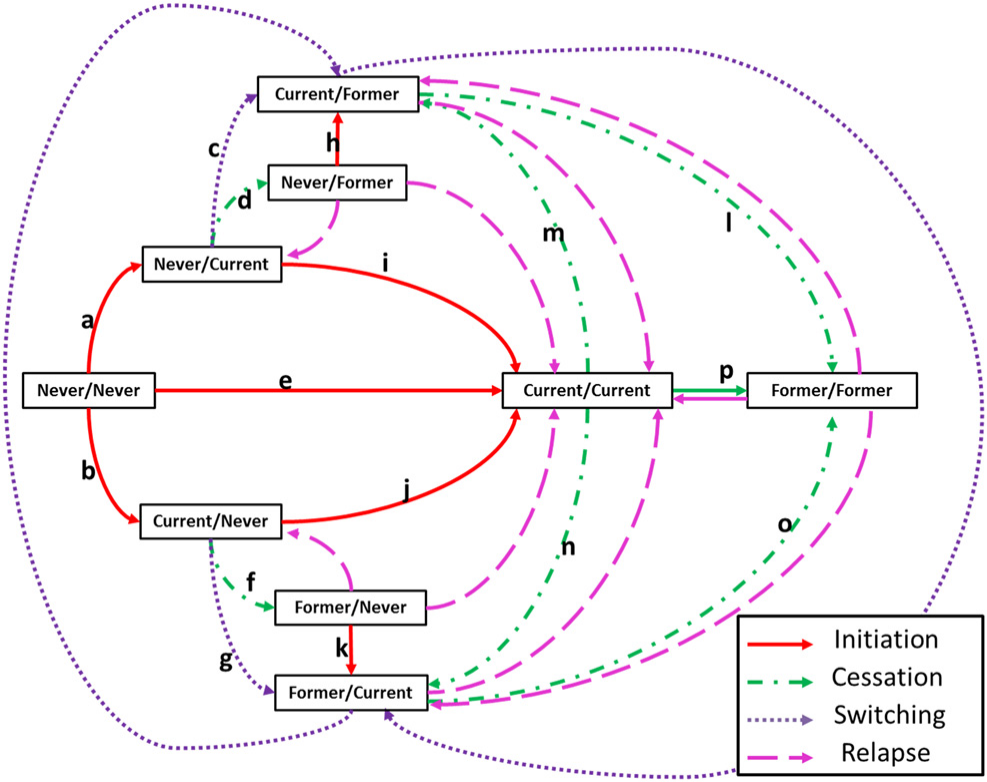
Tobacco use status transitions for a two product model formulation. Transition behaviors (illustrated by directed edges) are categorized into four groups: initiation, cessation, switching, and relapse. Nine possible use states are represented as boxes in which the first and second terms correspond to use of the first and second product, respectively.

The full set of product use states and transitions for a two-product model formulation are represented in Fig. 1. The number of members of a population subgroup who change tobacco use states is calculated as a function of sex, age, and current and past use of tobacco products. The number of members of a subgroup who survive or die is a function of sex, age, and tobacco use status, including time since cessation for former users.

### Mathematical Implementation

The model is implemented as a discrete dynamical systems model that tracks the number of persons in the population and its constituent subpopulations over time. Each subpopulation is defined by a unique combination of sex, age, and tobacco product use. Age and time are characterized by single year, although this aspect of the model can be modified if desired. The members of each subpopulation have a specified probability of dying and set of probabilities for transitioning from one tobacco use state to another. At each discrete time step in a simulation, the model updates the number of members of each subpopulation by calculating the number of individuals that transition to the subpopulation and remain alive during the time step. The size of the subpopulation is also updated by births and net international migration. For a particular subpopulation, this calculation is represented by the following equation:

$$x_{i}\left(t+1\right)\;=\;x_{i}\left(t\right)-x_{i}^{out}\left(t+1\right)+x_{i}^{in}\left(t+1\right)+b_{i}\left(t+1\right)+m_{i}\left(t+1\right)$$

where

- *x*_*i*_ *(t)* is the number of people in subpopulation *i* at time *t*.
- *x*_*i*_^*out*^ *(t+1)* is the number of people that leave subpopulation *i* between time *t* and *t+*1, due to a change in tobacco product usage, death, or aging. (Because age is one of the attributes used to define a subpopulation, *x*_*i*_ *(t)* is necessarily equal to *x*_*i*_^*out*^ *(t+1)* since an individual either ages, and hence leaves the subpopulation, or dies, and also leaves the subpopulation).
- *x*_*i*_^*in*^ *(t+1)* is the number of people that join subpopulation *i* and do not die between time *t* and *t+*1.
- *b*_*i*_ *(t+1)* is the number of births into subpopulation *i* between time *t* and *t+*1.
- *m*_*i*_ *(t+1)* is the net international migration into subpopulation *i* between time *t* and *t+*1.

Given the large number of subpopulations often represented in the model, the following vector-based, difference equation is used to update all subpopulations simultaneously:

$$x\left(t+1\right)\;=\;A\left(t+1\right)x\left(t\right)+b\left(t+1\right)+m\left(t+1\right)$$

where

- ***x*** *(t)* is a vector of subpopulation sizes. The *i* ^th^ entry is the number of individuals in subpopulation *i* at time *t*.
- ***A*** *(t+1)* is a matrix that determines deaths and product use transitions between subpopulations. The entry in the *i* ^th^ row and *j* ^th^ column is the fraction of subpopulation *j* that transitions to subpopulation *i* and does not die between times *t* and *t+*1.
- ***b*** *(t+1)* is a vector containing the number of births that occur between times *t* and *t+*1. The *i* ^th^ entry contains the number of individuals born into subpopulation *i* between times *t* and *t+*1.
- ***m*** *(t+1)* is a vector containing the net number of international migrants between times *t* and *t+*1. The *i* ^th^ entry contains the net number of individuals migrating into or out of subpopulation *i* between times *t* and *t+*1. This entry is positive when immigration exceeds emigration and is negative when emigration exceeds immigration.

S1 Appendix provides additional mathematical details about the calculations.

### Status Quo Scenario Data Inputs and Assumptions

In the status quo scenario, cigarettes are the only tobacco product being modeled. This section presents a brief summary of the input parameters and values used to project this scenario. S2 Appendix provides a complete explanation of the parameter values and their sources.

#### Initial Population

The simulation begins with an initial population that reflects the sex, age, and cigarette smoking distribution (i.e., never, current, and former smokers [including time since cessation]) of the US population in 2000 [46–47]. The population size by sex and age come from US Census Bureau estimates [46]. Cigarette smoking prevalence by sex, age, and time since cessation for adults ages 18 years and over come from estimates from NHIS data for 2000 [47].

#### Births and Net International Migration

Projected births and net international migration in the simulation are derived from projections of birth, net international migrants, and population size produced by the US Census Bureau for the US population from 2001–2050 [46]. Smoking prevalence for immigrants at the time of arrival to the US is obtained from NHIS smoking prevalence estimates for recently arrived immigrants [47].

#### Deaths

Never smoker death rates form the basis of projections of mortality and tobacco-attributable mortality in the simulation. US death rates from vital statistics data are used for never smoker death rates at baseline in 2000 for ages less than 35 years [48], given that tobacco-attributable mortality is assumed to be minimal prior to this age [49]. Never smoker death rates for ages 35 years and over are estimated from NHIS—Linked Mortality Files (NHIS-LMF) data [50]. NHIS-LMF never smoker death rates are adjusted for low mortality in the NHIS’s civilian non-institutionalized population using the ratio of US death rates from vital statistics data to NHIS-LMF death rates by sex and age [51–55]. Never smoker death rates are projected for the period from 2000–2050 using mortality scaling factors obtained from the Lee-Carter mortality forecasting method [56]. The resulting projected, adjusted never smoker death rates are then converted to probabilities of dying by sex and age using standard demographic methods [57]. These probabilities of dying are multiplied by relative risks according to smoking status to obtain mortality risks for current and former smokers. The relative risks are estimated as hazard ratios using Cox proportional hazard models with NHIS-LMF data from 1997–2004 NHIS participants followed for mortality through the end of 2006 [50]. The final projected probabilities of dying by smoking status are then multiplied in the model by the numbers of members of the relevant population subgroups to obtain the numbers of individuals surviving and dying during the time step. S2 Appendix contains a complete list and explanation of the data sources that were used to compile and produce the mortality statistics that are used in the model. For example, Tables E through L in S2 Appendix present death rates and hazard ratios by smoking status estimated from NHIS-LMF data and mortality scaling factors obtained with the Lee-Carter method, and Figs. D and E in S2 Appendix show the sex- and age-specific smoking initiation and cessation rates that are used in the model.

#### Smoking Initiation and Cessation

Age-specific smoking initiation and cessation rates are obtained from smoking histories for birth cohorts reconstructed from NHIS data by CISNET researchers [33]. The rates are selected from the most recent cohorts with data available from the late 1990s and are available on the CISNET website [58]. In the status quo scenario, age-specific initiation and cessation rates are assumed to remain constant throughout the projection period. Initiation rates represent established cigarette use based on having smoked at least 100 cigarettes in one’s life. Cessation rates reflect successful smoking cessation for at least two years; therefore, transition probabilities for relapse behaviors are set to zero in the modeling simulations presented here. CISNET estimates from NHIS data are also used to estimate cigarette use prevalence for ages less than 18 years in the model’s initial population in 2000 [33].

### Hypothetical Scenario Inputs and Assumptions

We also examined a set of scenarios related to the introduction of a hypothetical new tobacco product. These scenarios are purely hypothetical in nature, and any model results presented here are not intended as accurate projections of future tobacco product use and harm. Instead, these scenarios were designed to demonstrate the functionality and capabilities of the model in general. The parameter values used to simulate these scenarios are shown in Table 1. This analysis assumes that a new product is introduced in the fourth year of the projection period, has a mortality risk that is lower than cigarette smoking, and results in changes in tobacco use behavior in current smokers and never smokers.

**Table 1:** Hypothetical Scenario Input Parameters and Values.

Parameter	Base Value	Range
Excess relative risk factor—represents new product risk as a proportion of excess relative risk for cigarette smoking	0.25	[0.01,0.50]
Proportion of cigarette smokers who switch to new product use on an annual basis	0.015 (A)*	[0,0.03]
	0.010 (B)**	
Proportion of cigarette smokers who transition to dual product use on an annual basis	0.015 (A)*	[0,0.03]
	0.020 (B)**	
Proportion of switchers and dual users coming from smokers who would have otherwise quit smoking that year	0.25	[0,0.5]
New product initiation factor—represents the new product initiation rate among never smokers as a proportion of the cigarette smoking initiation rate	0.5	[0.25,0.75]
Proportion of new product initiates who would have otherwise initiated cigarettes that year	0.5	[0.25,0.75]
Proportion of new product users who switch to cigarette use on an annual basis	0.05	[0,0.1]
Proportion of new product users who transition to dual product use on an annual basis	0.05	[0,0.1]

* Hypothetical Scenario A, in which transition rates to dual use and switching are equivalent

** Hypothetical Scenario B, in which transition rate to dual use is greater than transition rate to switching

Among current smokers, the introduction of the new product causes some to switch from cigarettes to the new product (transition **g** in Fig. 1) and some to become dual users (transition **j**). Switching has the effect of decreasing the number of cigarette smokers; however, it may increase the number of overall tobacco users given that some individuals who would have otherwise quit smoking (transition **f**) may switch to the new product. Similarly, dual use may increase the number of cigarette smokers because some of these dual users may have otherwise quit smoking in the absence of the new product. Two hypothetical scenarios are examined. In Hypothetical Scenario A, the transition rates from current smoking to dual use and switching are equivalent. In Hypothetical Scenario B, the transition rate from current smoking to dual use is greater than the transition rate for switching. The specific values for these two scenarios are shown in Table 1.

Among never smokers, the introduction of the new product may cause some individuals who had not previously smoked cigarettes to initiate the new product (transition **a**). Some new initiates may have otherwise taken up smoking (transition **b**), so the new product could have the effect of reducing smoking initiation. Conversely, for some new initiates, use of the new product may lead to cigarette use among those who would have otherwise not become cigarette smokers (transitions **c** and **i**).

Similar to rates for cigarette smoking, initiation rates for the new product represent established use and cessation rates reflect successful cessation that has been sustained for at least two years.

The hypothetical new product is assumed in these scenarios to have lower all-cause mortality risk than cigarettes. Relative risks for current dual users are set equal to the maximum of the relative risks for the individual products. Individuals who switch from cigarette smoking to the new product are assumed to have lower mortality risk than current smokers, but higher risk than former smokers who quit use of tobacco products entirely. Section S2.2 in S2 Appendix contains more detailed information about these risk calculations.

### Sensitivity Analysis

Sensitivity analyses were performed for Hypothetical Scenario A using the input parameter ranges shown in Table 1. Parameter sweeps are used to illustrate effects of uncertainty from a single or multiple variables and a Monte Carlo simulation using Latin hypercube sampling is conducted for multivariate uncertainty analysis. Parameter sweeps provide information on the sensitivity of estimates to variability in one or a few key parameter values, whereas the multivariate analysis illustrates the cumulative effect of uncertainty for all of the parameter values.

For parameter sweeps, deciles of the ranges in Table 1 are used, assuming a uniform distribution and holding all other parameter values constant. For multivariate uncertainty analyses, uniform distributions are assumed across the ranges for input parameters provided in Table 1. One thousand simulations were conducted and the mean, median, 2.5^th^, 25^th^, 75^th^, and 97.5^th^ percentiles of the output distribution are presented.

### Model Projections

Product use prevalence and mortality are the two primary outputs of interest in the scenario analyses. The primary mortality statistic of interest is the cumulative difference in deaths between the status quo and hypothetical new product scenarios. If *d*_*1*_ *(i*,*a)* and *d*_*2*_ *(i*,*a)* denote the annual number of deaths (in year *i* for individuals with age *a*) for the status quo and new product scenarios, respectively, then the cumulative difference in deaths between the scenarios in year *Y* for individuals between ages *a1* and *a2* (inclusive) is

$$\sum_{i=0}^{Y}\sum_{a=a1}^{a2}\left[d_{2}\left(i,a\right)-d_{1}\left(i,a\right)\right]$$

In the following sections, we compare the cumulative number of deaths between ages 35 to 84 years in the two scenarios. This metric reflects differences in survivorship through age 85 between the scenarios due to tobacco-attributable mortality, given that we assume no difference in mortality risks by tobacco use status for ages less than 35. Changes in premature mortality between the two scenarios are thus here defined as the difference in survivorship through age 85, although other measures of premature mortality such as cumulative differences in total tobacco-attributable deaths can be easily calculated in the model as well.

We also divide each new product scenario into two subscenarios to analyze which factors may be causing an overall benefit or harm to the population. In the first subscenario, we assume that the only pathway to using the new product is for current smokers to switch to the new product or to become a dual user of both cigarettes and the new product. New product initiation among never smokers is not permitted in this subscenario, which allows for observation of the impact of the new product on the population of smokers. The second subscenario allows for observation of the impact of the new product on the population of never smokers by assuming that the only pathway to use of the new product is initiation among never smokers. Switching from cigarettes to the new product is not permitted in this subscenario.

### Model Specifications

The model has been implemented in MATLAB version 8.1.0.604 (R2013a).

## Results

### Model Evaluation and Validation

**Fig 2:**
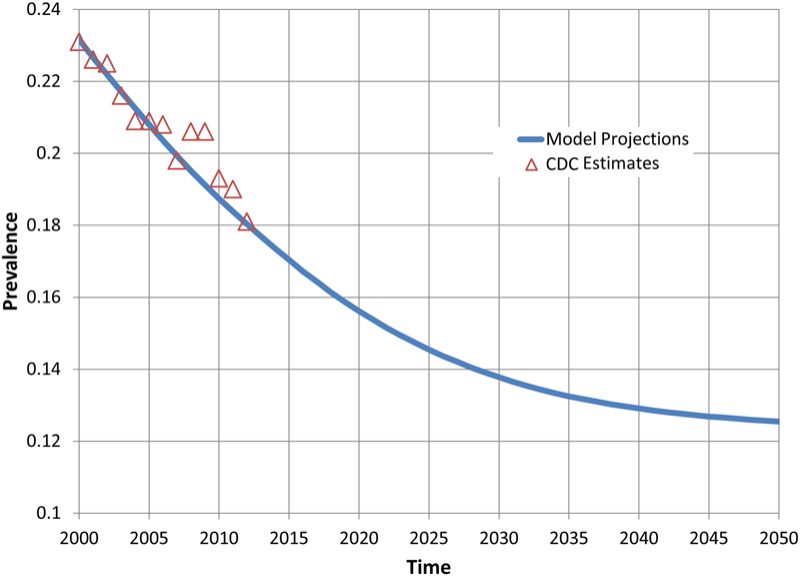
Observed and projected US adult current cigarette smoking prevalence, 2000–2050.

**Fig 3:**
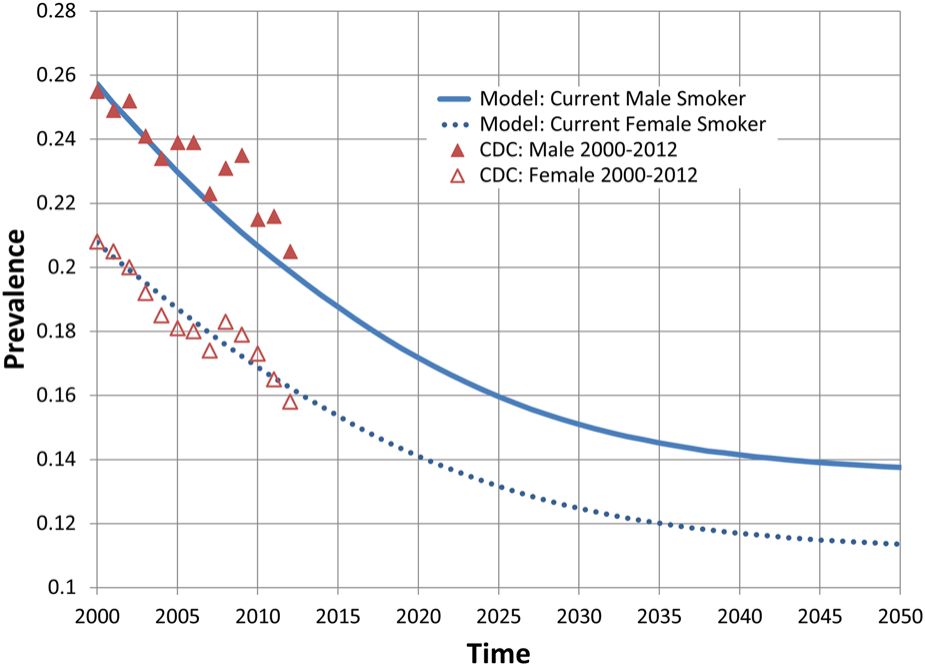
Observed and projected US adult current cigarette smoking prevalence by sex, 2000–2050.

To aid in model evaluation and validation, we present comparisons of results from our model and published estimates and projections for the US population. Full details of these comparisons are provided in S3 Appendix and are summarized briefly here. We first compare US population and mortality projections from our model with US Census Bureau projections for the period from 2000 to 2050 [59,60]. Census and model projections of the US population are within 1% of one another for each year of the projection period (Fig. A in S3 Appendix). Model results for annual total deaths in the US are generally consistent with Census projections throughout the projection period (Fig. B in S3 Appendix). Model projections of overall adult smoking prevalence and adult prevalence by sex for the period of 2000 to 2012 are similar to published estimates from the Centers for Disease Control and Prevention (CDC) that use NHIS data [47] and show a similar pattern of decline (Figs. 2 and 3). Model projections of smoking prevalence by age group for years 2000, 2005 and 2010 are also generally consistent with published CDC estimates (Table A in S3 Appendix).

The model can also be used to estimate smoking-attributable mortality for the US population. Most estimates of smoking-attributable mortality for the US fall within the range of 400,000 to 500,000 deaths per year [1,61–63]. Our model estimates an average of 416,000 annual smoking-attributable deaths in the US from 2000–2004, which is consistent with the CDC’s estimate of 392,000 annual smoking-attributable deaths (excluding deaths from secondhand smoke, fires, and perinatal causes) during this period. S3 Appendix presents additional information about the various methods used to estimate smoking-attributable mortality in the US.

### Hypothetical Scenario Results

**Fig 4:**
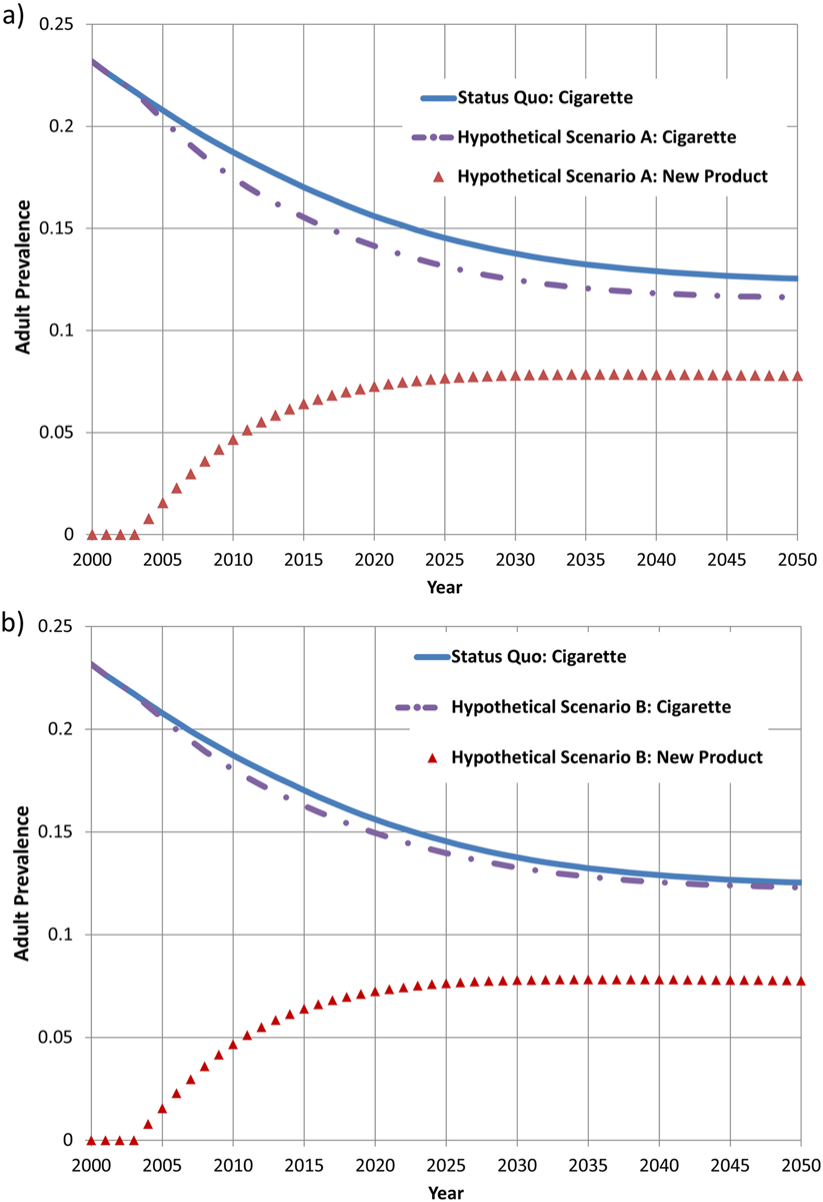
Comparison of cigarette smoking and new product use prevalence in status quo and hypothetical scenarios: (a) hypothetical scenario A, in which annual transition rates among smokers are 1.5% for dual use and switching (b) hypothetical scenario B, in which annual transition rates among smokers are 2% for dual use and 1% for switching.

Fig. 4 presents model projections of tobacco use prevalence, comparing the status quo cigarette-only scenario with the two hypothetical two-product scenarios described previously. All scenarios and results are designed to demonstrate the capabilities of the model and thus are purely hypothetical and are not intended to represent actual or expected products or behaviors. In Hypothetical Scenario A, in which transition rates to dual use and switching are equivalent, adult smoking prevalence declines by approximately 1.5% relative to the status quo scenario by 2020 (Fig. 4a), a decline that is driven primarily by product switching among current smokers. The difference in adult smoking prevalence between the status quo and hypothetical scenarios begins to decrease and is less than 1% by 2050, which is likely due to youths who initiated new product use earlier in the simulation having switched to cigarettes. At the same time, the prevalence of new product use among adults increases until it reaches a steady state of approximately 8% after about 20 years of the simulation. In Hypothetical Scenario B, in which smokers transition to dual use at a higher rate than smokers switch to the new product, changes in adult smoking prevalence are more modest with the difference in adult smoking prevalence between the status quo and hypothetical scenarios being less than 1% in 2020 and less than 0.25% in 2050 (Fig. 4b). These differences are smaller than in Hypothetical Scenario A because fewer individuals are stopping use of cigarettes entirely in switching to the new product and more individuals are instead using the new product in addition to continuing use of cigarettes.

**Fig 5:**
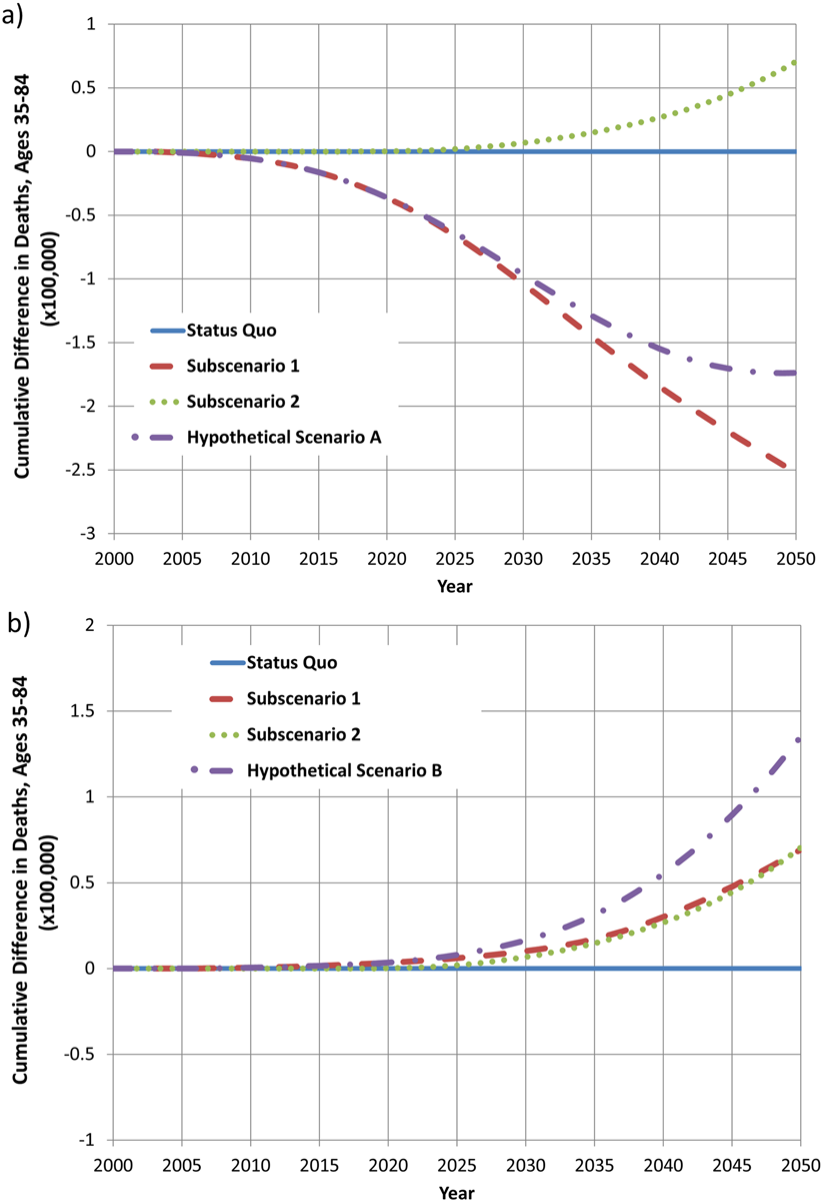
Comparison of cumulative deaths, ages 35–84, in status quo and hypothetical scenarios: (a) hypothetical scenario A, in which annual transition rates among smokers are 1.5% for dual use and switching (b) hypothetical scenario B, in which annual transition rates among smokers are 2% for dual use and 1% for switching.

The effects of Hypothetical Scenarios A and B on cumulative premature deaths relative to the status quo scenario are shown in Fig. 5. Premature mortality is here defined as deaths prior to age 85, as explained in the Methods section. These results are also shown for each of the two subscenarios discussed previously. Subscenario 1 assumes that the only pathway to new product use is for current smokers to switch to the new product or become a dual user; new product initiation is not permitted among never smokers. Subscenario 2 assumes that the only pathway to use of the new product is initiation among never smokers; switching from cigarettes to the new product is not permitted.

In Hypothetical Scenario A, a steady reduction in premature deaths is observed (Fig. 5a), driven primarily by the decline in mortality risk for current smokers who switched to the new product. After approximately 25 years, an increase in premature deaths is observed among new product initiates, some of whom have transitioned to cigarette smoking, thus counteracting the population health benefits from product switching. This impact on premature mortality is not observed for several decades, given that it takes time for new product initiates to reach the ages at which excess mortality from tobacco use generally occurs. In Hypothetical Scenario B, an increase in premature deaths relative to the status quo scenario is observed in the scenario overall and in each of the subscenarios (Fig. 5b). This mortality increase is observed despite a slightly lower smoking prevalence in the hypothetical scenario. The mortality increase occurs because the decrease in mortality due to slightly lower smoking prevalence in this scenario is more than offset by the increases in mortality due to new product initiation among individuals who would not have initiated tobacco use in the absence of the new product and increases in mortality due to dual product use among those who otherwise would have quit tobacco use in the absence of the new product.

### Impact of Varying Product Risk, Switching, and Initiation

**Fig 6:**
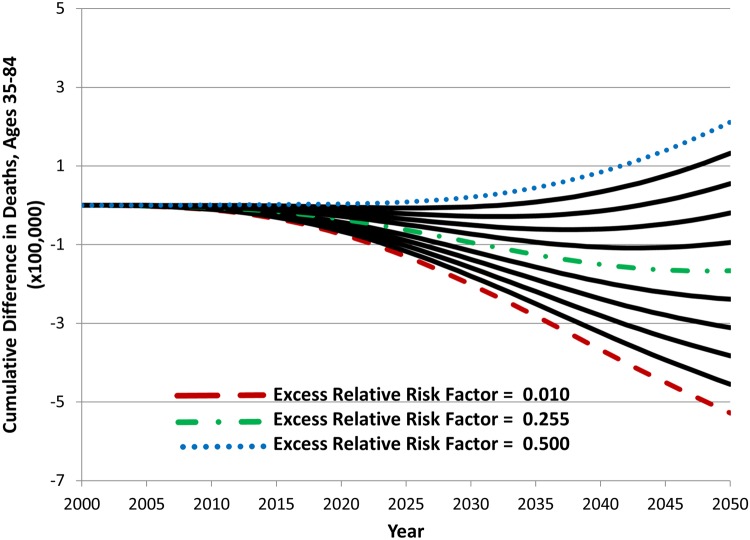
The effect of new product excess relative risk factor values on cumulative difference in deaths between the status quo and hypothetical scenario A, 2000–2050.

Fig. 6 shows the results of a parameter sweep across a range of excess relative risk factor values for the new product. New product excess relative risks are varied from 1% to 50% of the excess risk of cigarettes. In these simulations, all other input parameter values are set to the base values listed in Table 1 (Hypothetical Scenario A) and remain constant. The comparison continues to be cumulative difference in deaths between the hypothetical and status quo scenarios. In the year 2050, cumulative difference in deaths with the hypothetical scenario changes from negative to positive at a new product mortality excess relative risk of about 35% of the risk of cigarettes, and cumulative difference in deaths at this point is increasing over time. For higher excess relative risk factors, an increased number of cumulative deaths is observed at earlier times.

**Fig 7:**
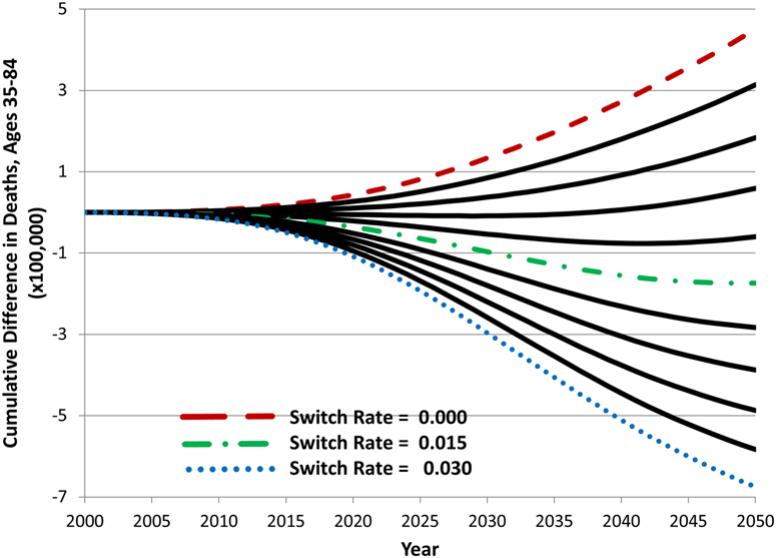
The effect of switching rate values on cumulative difference in deaths between the status quo and hypothetical scenario A, 2000–2050.

Fig. 7 presents the results of a single parameter sweep for the annual rate of switching from cigarettes to the new product, from 0 to 3% per year. Holding all other parameter values constant at their base values, increasing the switching rate tends to decrease cumulative number of deaths in the population. It should be noted, however, that not all switching rates result in a reduction of deaths and that with other input parameters set to their hypothetical base values the switching rate value must exceed 1% annually to produce a decrease in cumulative deaths. This result occurs because of the effects of other parameter values such as those for initiation of the new product, transition from the new product to cigarettes, and the proportion of smokers switching to the new product who would have otherwise quit use of tobacco products entirely.

**Fig 8:**
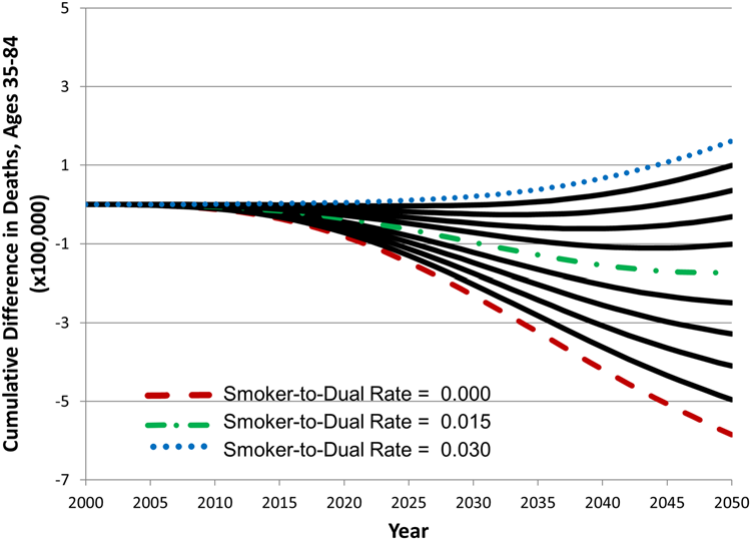
The effect of changes in smoker to dual use transition rate values on cumulative difference in deaths between the status quo and hypothetical scenario A, 2000–2050.

Fig. 8 presents the results of a single parameter sweep for the annual rate of cigarette smokers transitioning to dual product use, from 0 to 3% per year. Holding all other parameter values constant at their base values, an increase in this value produces an increased number of cumulative deaths. With other parameters set to their hypothetical base values, an annual rate of current smokers transitioning to dual use at or below 2.4% results in a cumulative reduction in deaths compared with the status quo scenario, and a rate above 2.4% results in a cumulative increase in deaths.

**Fig 9:**
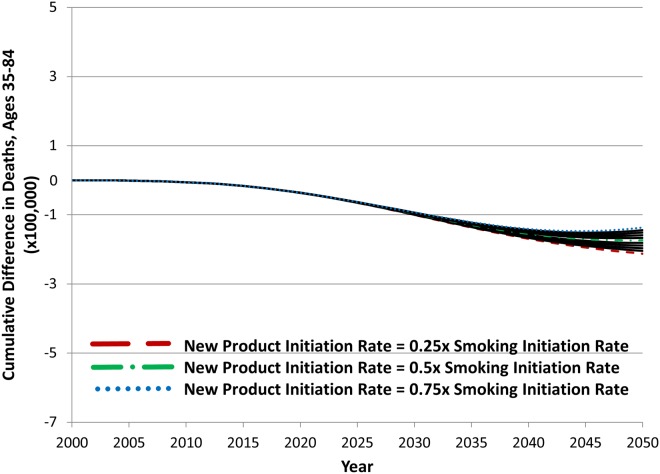
The effect of new product initiation rate values on cumulative difference in deaths between the status quo and hypothetical scenario A, 2000–2050.

Fig. 9 illustrates the impact of varying the new product initiation rate from 25% to 75% of the cigarette smoking initiation rate. As expected, because of the lag between initiation and increased mortality risk, varying this parameter value has little impact on premature deaths until decades after introduction of the new product, and consequently cumulative difference in deaths is less sensitive to this value than it is to the excess relative risk factor and switching rate values over the time period. For higher initiation rate values, cumulative difference in deaths starts to increase in the later years of the simulation.

### Bivariate and Multivariate Sensitivity Analyses

**Fig 10:**
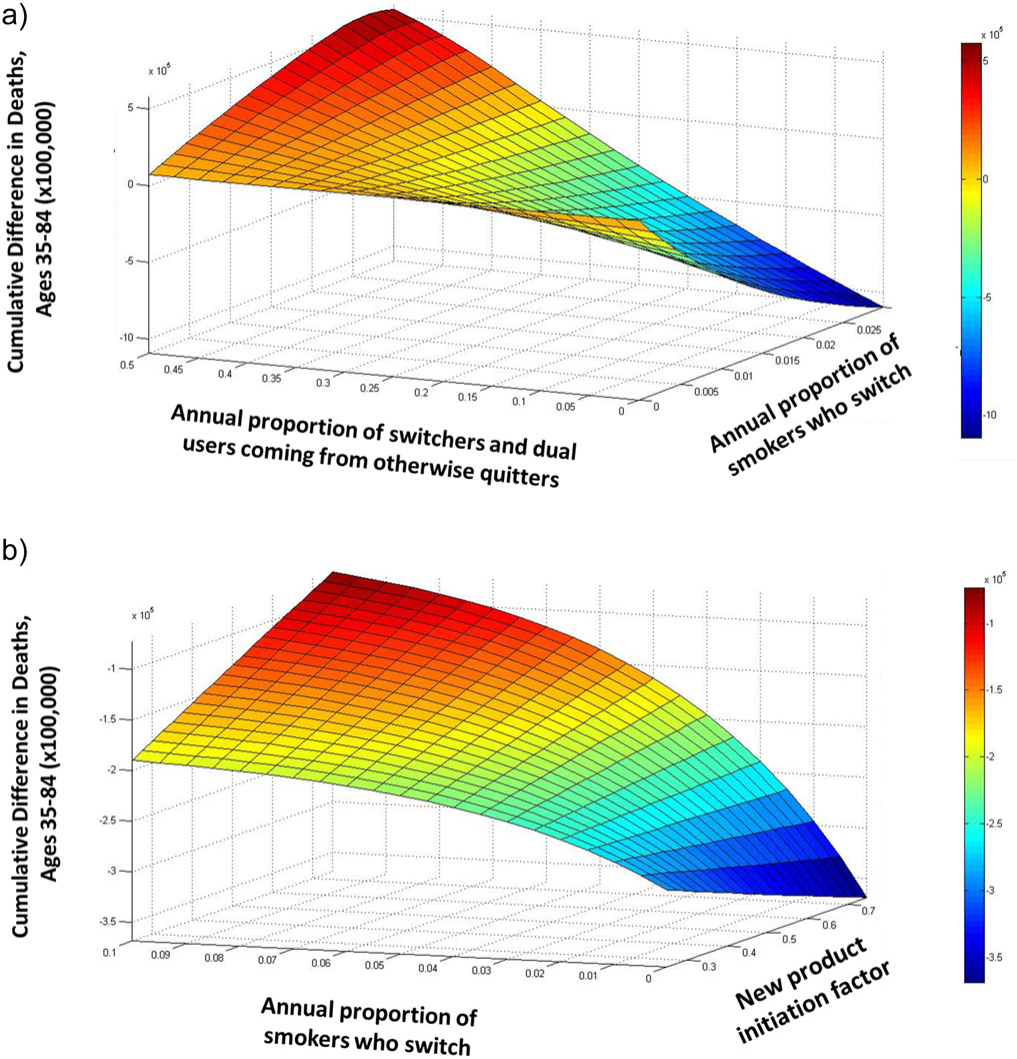
Bivariate parameter sweeps of (a) annual proportion of cigarette smokers switching to new product and the proportion of these switchers and dual users who would have otherwise quit smoking that year (b) new product initiation factor and annual proportion of new product users switching to cigarettes or transitioning to dual use on cumulative difference in deaths for ages 35–84 between the status quo and hypothetical scenario A in 2050.

Fig. 10 presents estimates of the mortality effects of varying two sets of parameters values for Hypothetical Scenario A while other parameter values are held constant. Fig. 10a presents projections of differences in cumulative deaths in 2050 as a function of the annual proportion of cigarette smokers who switch to the new product and the proportion of new switchers and dual users who would have otherwise quit smoking that year. These results show that with other parameters held constant at their base values, an increase in the rate of smokers switching to the new product tends to lower cumulative deaths, and an increase in the proportion of these smokers who would have otherwise quit smoking that year tends to increase deaths. Fig. 10b shows cumulative deaths in 2050 as a function of the new product initiation factor and the annual proportion of new product users switching to cigarettes and transitioning to dual use. With other factors held constant, these results show a general reduction in cumulative deaths that decreases with higher levels of new product initiation and new product initiates transitioning to cigarette use.

**Fig 11:**
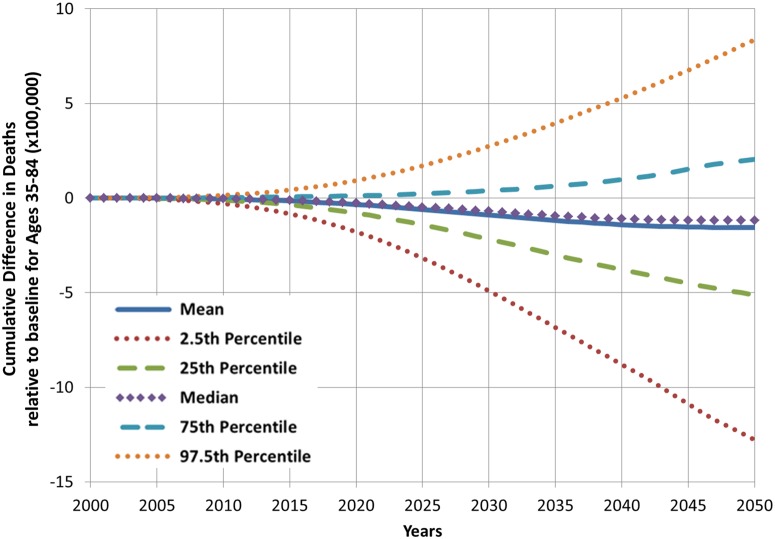
Projected cumulative difference in deaths for the new product scenario compared with the status quo scenario, from multivariate uncertainty analysis, 2000–2050.

**Fig 12:**
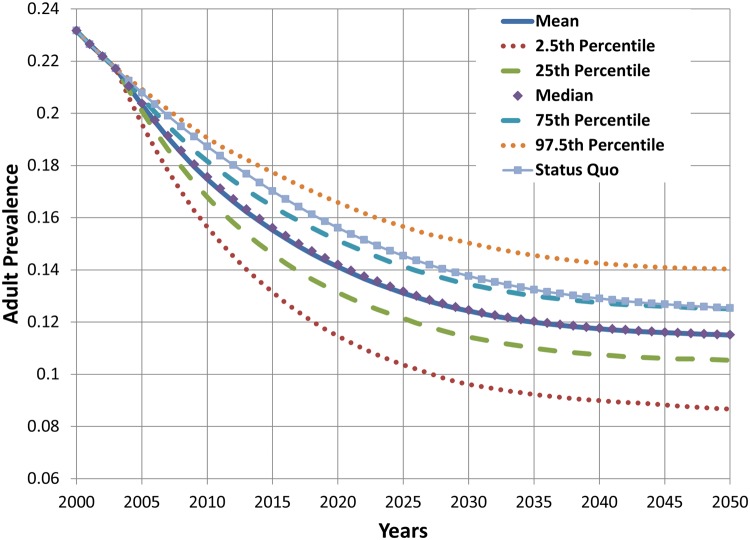
Projected adult prevalence of cigarette smoking from multivariate uncertainty analysis, 2000–2050.

**Fig 13:**
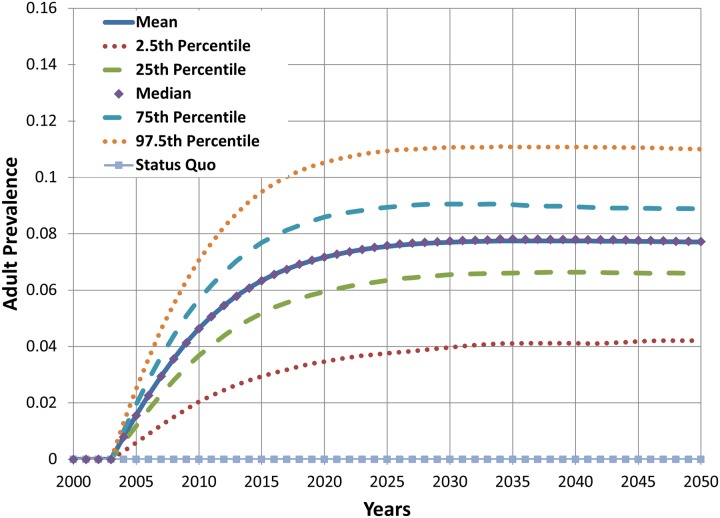
Projected adult prevalence of new product use from multivariate uncertainty analysis, 2000–2050.

Figs. 11, 12, and 13 show the results from multivariate uncertainty analyses in which all of the parameters in Table 1 were assigned uniform distributions, and values for these parameters were sampled using Latin hypercube sampling. The figures show the results from 1,000 simulations. Fig. 11 shows that the mean estimate of cumulative difference in deaths for the hypothetical product scenario is slightly negative over time, but the region spanned by the 25^th^ and 75^th^ percentile projections include both negative and positive cumulative differences in death. The figure also shows that projections become highly uncertain when accounting for variation in all of the parameters, and that uncertainty grows with the length of the projection period. Results shown in Fig. 12 indicate that adult cigarette smoking prevalence in 2050 is lower than the status quo scenario value in approximately 75% of the simulations with varying hypothetical parameter input values. Results presented in Fig. 13 show that for the given parameter values and distributions the projected prevalence of new product use in 2050 has a mean value of about 8% and falls between 4% and 12% in 95% of the simulations with varying parameter input values.

## Discussion

In this study, we have presented a dynamical systems model for assessing the impacts of multiple tobacco products on population use and health. This model significantly adds to the developing work on models that analyze the population effects of tobacco products. We have demonstrated the ability of the model to project demographic trends, smoking prevalence, and smoking-attributable mortality for the US population overall and by sex and age group that are consistent with empirical evidence and projections developed by the CDC and Census Bureau. We have also simulated the impact of a hypothetical new lower-risk tobacco product on tobacco use prevalence and premature mortality using a range of parameter input values to identify the factors that influence population health. In doing so, we have highlighted the parameter values and conditions under which mortality may increase or decrease in the population. Although this analysis examined the effects of the introduction of a hypothetical new tobacco product in the marketplace, this modeling approach is also applicable to examining the effects of policies and measures that affect a tobacco product or class of products and that may result in changes in product use behaviors such as initiation, dual use, or switching.

This study has demonstrated the sensitivity of model estimates and projections to uncertainty and variation in parameter input values. As the various parameter value sweeps have indicated, reasonable ranges for input values can cause substantive differences in model projections over time. Moreover, incorporation of the full range of uncertainty for all of the relevant parameter values can produce a wide range of possible model estimates.

Our analyses also demonstrate the importance of the timeframe being examined. For example, in Hypothetical Scenario A, a reduction in deaths is observed during the first few decades as current smokers switch to a lower risk product. Increased initiation of the new product among never tobacco users is also observed, but because of the lag between initiation and increased mortality risk, it takes several decades before this increase in tobacco use produces noticeable increases in premature mortality. If the initiation rate and risk of the new product are high enough, then the potential mortality benefits from switching among smokers can be offset. Another challenge in projecting population health impacts over time is that the uncertainty of the estimates increases over time, as observed in our model projections.

These results and considerations illustrate the complexity involved in making determinations about the population health effects of new tobacco products. We have presented estimates for measures such as cumulative deaths avoided in the presence of a hypothetical new product, but analyses of the appropriate health metrics and time frames that should be used in the overall evaluation of the population health effects of a tobacco product go far beyond the scope of this study. In addition, the estimates presented here illustrate the degree of uncertainty associated with projections due to factors such as differences in parameter input values and the effect that this uncertainty can have on the interpretation of results. Finally, additional thought is needed concerning the magnitude and distribution of any estimated differences in health outcomes in a population, however such outcomes are defined, that would be considered evidence of a population health benefit due to use of a new tobacco product.

Although the scenarios examined in this study were purely hypothetical, they have provided a number of insights concerning the relationship between individual risk and overall population health impact. First, the risk of a new tobacco product clearly has a strong effect on the product’s impact on population health, as expected. Also important, however, are the behaviors associated with the new product such as product initiation and cessation as well as behaviors associated with other products such as cigarette smoking initiation and cessation. We have shown, for example, that the maximum mortality benefit from smokers transitioning to a new lower-risk product would occur if those who use the new product are smokers who are otherwise unwilling or unable to quit smoking and who switch completely to the new product. In this case, population smoking cessation rates increase due to product switching, and mortality is reduced as smokers switch to use of a less harmful product. On the other hand, the model estimates an increase in deaths during the projection period when transition rates to the new product among smokers are high and many of the smokers who use the new product would have otherwise quit use of tobacco products entirely.

A significant concern about the introduction of a new tobacco product is its potential for use by youth. Most tobacco use begins during adolescence and recent studies have found increases in use of some non-cigarette products among youth [6]. At the individual level, an increase in risk would occur if a never user who would otherwise not smoke cigarettes starts using the new tobacco product, and in particular if they then transition to cigarette smoking. A decrease in risk would be achieved if a never user who would have smoked cigarettes instead uses a lower-risk product and does not transition to smoking cigarettes. The overall impact on population health can be estimated by integrating the impact to tobacco users and non-users across the entire population. In this analysis, we have demonstrated the impact of use of a hypothetical new tobacco product on new tobacco users by presenting results from scenarios in which the proportion of new product initiates who otherwise would have smoked and the proportion of new product users who transitioned to cigarette smoking were allowed to vary. This analysis is particularly timely and relevant to FDA’s regulation of tobacco products. Under the Federal Food, Drug, and Cosmetic Act, FDA can authorize a new tobacco product to be marketed with a “reduced exposure” or “reduced risk” claim if it is expected to benefit the population as a whole (Sec 911) [9]. To make this determination, FDA is required to apply a population health standard that takes into account the increased or decreased likelihood that current tobacco users and non-users will use the product. Our results highlight the need for detailed and accurate data to evaluate the impact of new products coming to market as well as the value of multivariate sensitivity analyses with uncertainty estimates in identifying the factors that most affect population health.

Our model has several important strengths. In these simulations, we have accounted for future demographic changes by projecting age-specific changes in mortality rates and by using US Census Bureau projections of births and international migration. As a result, our population and mortality projections are closely aligned with Census projections of population size and deaths. The incorporation of demographic change in our model allows us to produce reasonable projections of tobacco-attributable mortality for the US over time, which has not generally been done previously. Levy and Friend [11], for example, projected smoking-attributable mortality for the US from 2000 to 2040, but they used demographic inputs from 1993 throughout the projection period. We have also used detailed smoking initiation and cessation rates by sex and age from recent cohorts, and our smoking prevalence projections are in close agreement with observed estimates for the US from NHIS data. Our modeling strategy thus allows us to reasonably project tobacco use and harm for a population over a specified period of time. In doing so, our approach may allow for more intuitive and readily interpretable results compared with models that project cohort life tables at a single point in time, such as the analysis presented by Bachand and Sulsky [45]. Our model is conceptually and mathematically flexible and can incorporate additional tobacco products, although increases in the number of products can present additional challenges in terms of data availability and uncertainty quantification. Our model can also represent a single cohort or a heterogeneous population that is reflective of a current or projected population, and can account for different types of product risk interactions such as synergism, antagonism, and independence. As a result, our approach is relevant not only to the analysis of introduction of a new tobacco product, but also to the evaluation of the impact of tobacco regulatory policies or other actions that may result in tobacco product users shifting from one class of products to another.

The model does have certain limitations. In the present study, we restricted the analysis to all-cause mortality. In the future, we intend to incorporate cause-specific mortality and morbidity to obtain a more complete characterization of the population health impact of tobacco products. Similarly, the relative risks currently used for cigarette smoking account for age, sex, smoking status, and time since quitting for former smokers, but they do not account for other measures of smoking exposure, such as number of years of smoking or average number of cigarettes smoked per day. As a result, we are not currently estimating the impact of changes in cigarette consumption among smokers over time. We also assume, as a simplifying assumption, that smoking initiation and cessation rates remain constant from the year 2000 forward in our projections. These rates do produce smoking prevalence estimates for the US that are extremely consistent with observed estimates during the first 13 years of the projection period. We also make certain assumptions about the combined mortality effects of multiple tobacco products due to limited data availability, including the assumption that dual use would not result in increased mortality risk compared to smoking alone. These assumptions can be refined when additional information on the mortality effects of multiple tobacco products become available. In fact, our model and its results demonstrate the need for further study of the combined effects of multiple tobacco products. Finally, the scenarios and estimates presented here are purely hypothetical in nature and may not reflect all of the possible behavior transitions that could affect population health. For example, the implementation of the model presented here uses smoking cessation rates that reflect established cessation for at least two years, and as a result, relapse to smoking is not modeled explicitly. It is possible, however, that the introduction of a new lower-risk tobacco product may have particular appeal for former smokers, some of whom may use the new product and eventually relapse to cigarette smoking, thus increasing their health risks. To address this issue, future model development should consider inclusion of explicit representation of relapse to smoking and other tobacco use, similar to the approach used by Carreras et al. [64] in their modeling study of smoking cessation rates in Italy. Similarly, initiation of the new product is allowed here through age 30, given that most tobacco use initiation occurs in youth and young adulthood, but it is possible that a new tobacco product could encourage use by individuals of older ages. Transitions such as smoking relapse and initiation of a new product at older ages could affect mortality trends and estimates, especially if the new product had particular appeal for former or never users of other tobacco products.

## Conclusion

This article has presented the implementation of a multiple-product, dynamical systems model to project tobacco use and harm for a population over time. We have demonstrated the capabilities of this model by projecting future cigarette smoking prevalence and smoking-attributable mortality and then simulating the effects of introduction of a hypothetical new lower-risk tobacco product. Using differing assumptions about product initiation, switching, dual use, and risk, we have demonstrated that potential mortality reductions from cigarette smokers switching to a new lower-risk product can be offset over time through increased initiation of the new product. We have also shown that the mortality effects of cigarette smokers transitioning to a lower-risk product are sensitive to the degree to which current smokers become dual users or switch completely to the new product and the extent to which uptake of the new product increases or decreases smoking cessation rates, as some smokers may switch to the new product instead of quitting use of tobacco products entirely. Finally, we have also shown that model projections can become imprecise when accounting for uncertainty across all input parameters and in projecting population effects over extended periods of time. These results underscore the need for accurate and detailed data for input parameter values and the importance of cautious and careful interpretation of model results.

In the future, the model can be expanded in a variety of ways. As has been noted, future model development can include the explicit representation of smoking relapse behavior with or without the introduction of new tobacco products, as such data become available. The model can also be expanded to track tobacco use and risks by demographic and socioeconomic characteristics such as race/ethnicity and education level in order to analyze the effect of tobacco use on health disparities and inequalities. Finally, the model can be expanded to include measures of morbidity, quality of life, and cause-specific mortality to more fully assess and analyze the population health effects and burden of tobacco products.

## Supporting Information

S1 Appendix Model Formulation. This appendix describes the conceptual and mathematical model details of the multi-state, dynamical systems population structure model that can be used to assess the effects of tobacco product use behaviors on population health.
(DOCX)

S2 Appendix Data Inputs and Assumptions. This appendix lists sources for population and cigarette smoking data that are used for the status quo model scenarios. The appendix also describes how input parameters were developed for the new product scenarios and relevant modeling assumptions.
(DOCX)

S3 Appendix Model Diagnostics and Validation. This appendix contains the results of model validation activities. Model projections for US population, cigarette smoking prevalence, and smoking attributable mortality are compared to published estimates from the US Census Bureau and US Centers for Disease Control and Prevention.
(DOCX)
