# Supplementary material for: Modeling the Potential Effects of New Tobacco Products and Policies: A Dynamic Population Model for Multiple Product Use and Harm
Source: PLoS One. 2015 Mar 27;10(3):e0121008. doi: 10.1371/journal.pone.0121008 (PMC4376806; doi:10.1371/journal.pone.0121008)
Supplement: S1 Appendix — This appendix describes the conceptual and mathematical model details of the multi-state, dynamical systems population structure model that can be used to assess the effects of tobacco product use behaviors on population health. (DOCX) [file pone.0121008.s001.docx]

**S1 Appendix: Model Formulation**

# S1.1 Conceptual Model Description

This section details the conceptual formulation for the dynamic, multi-state model for assessing the effects of tobacco product use behaviors on population health. Up to any combination of *N* tobacco products are being consumed within a specified population, and the model can be used to track the number of people in subpopulations over time. Subpopulations are defined to be groups of individuals that share a common set of the following attributes:

- Age, denoted by *a =* 0, 1, 2,…, *G,G^+^*. *G* represents the maximum individual age tracked and *G^+^* represents the group of ages greater than *G*. For example, model formulations frequently set *G* = 99 and *G^+^* represents *a* > 99.
- Sex, denoted by *s = male*, *female*.
- Tobacco product use status. An individual’s tobacco product use status with respect to the *N* tobacco products is denoted with an *N*-tuple, $u=\left(u_{1},u_{2},...,u_{N}\right)$, where $u_{k}$, *k =* 1,…, *N*, is be defined to be

$$u_{k}=\left\{\begin{matrix} n,\text{ never user of product }k \\ c,\text{ current user of product }k \\ f_{j},\text{ former user of product }k\text{ who quit }j\text{ years ago, }j=1,\text{ }2,\text{ }...,M_{k} \end{matrix}\right.$$

where *M_k_* denotes the maximum number of years tracked for quitters of product *k*. This manuscript uses the notation $u_{never}$to denote the tobacco product use status of never use for all *N* products.

Over time, individuals age during each modeled time step; individuals may die; and individuals may change their tobacco product use status, e.g., they may initiate new products, quit using products, relapse and take up products they had previously quit, and so on. Changes in age and tobacco use status cause individuals to move from one subpopulation to another. The probability that an individual transitions from one tobacco use status to another is determined by the individual’s current age, sex, and tobacco use status. Fig. A and B show the set of tobacco-use-status transitions for one- and two-product model formulations, respectively. This model can be extended to include additional products.


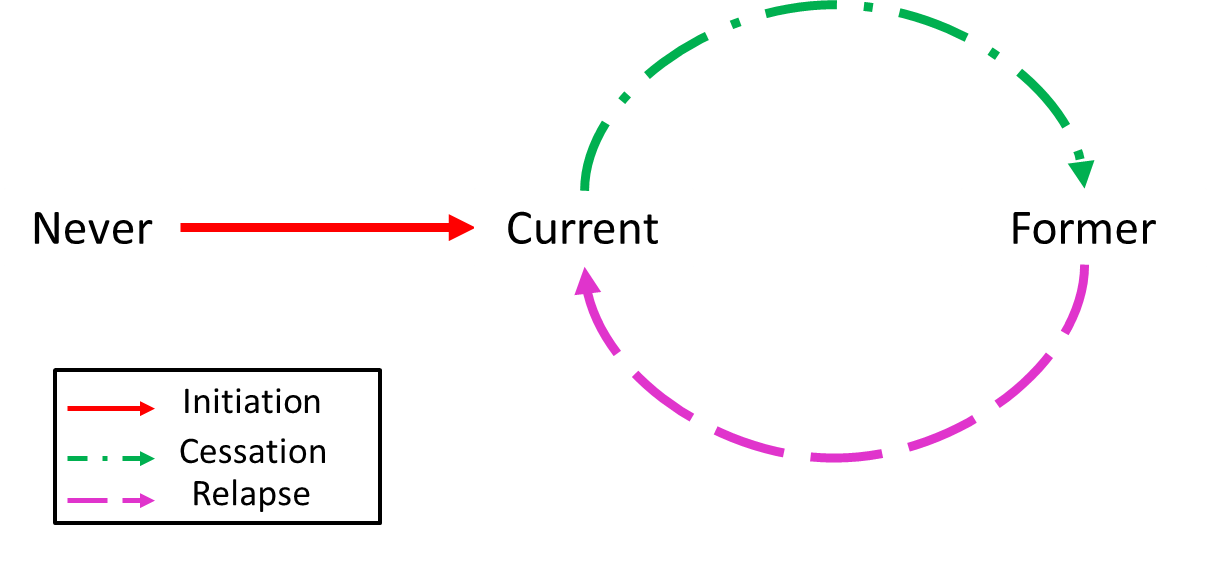


**Figure A: Tobacco-use-status transitions for a single product model formulation.**

**Figure Note**: In the current model transition takes place among subpopulations defined by age, sex, and tobacco use status.


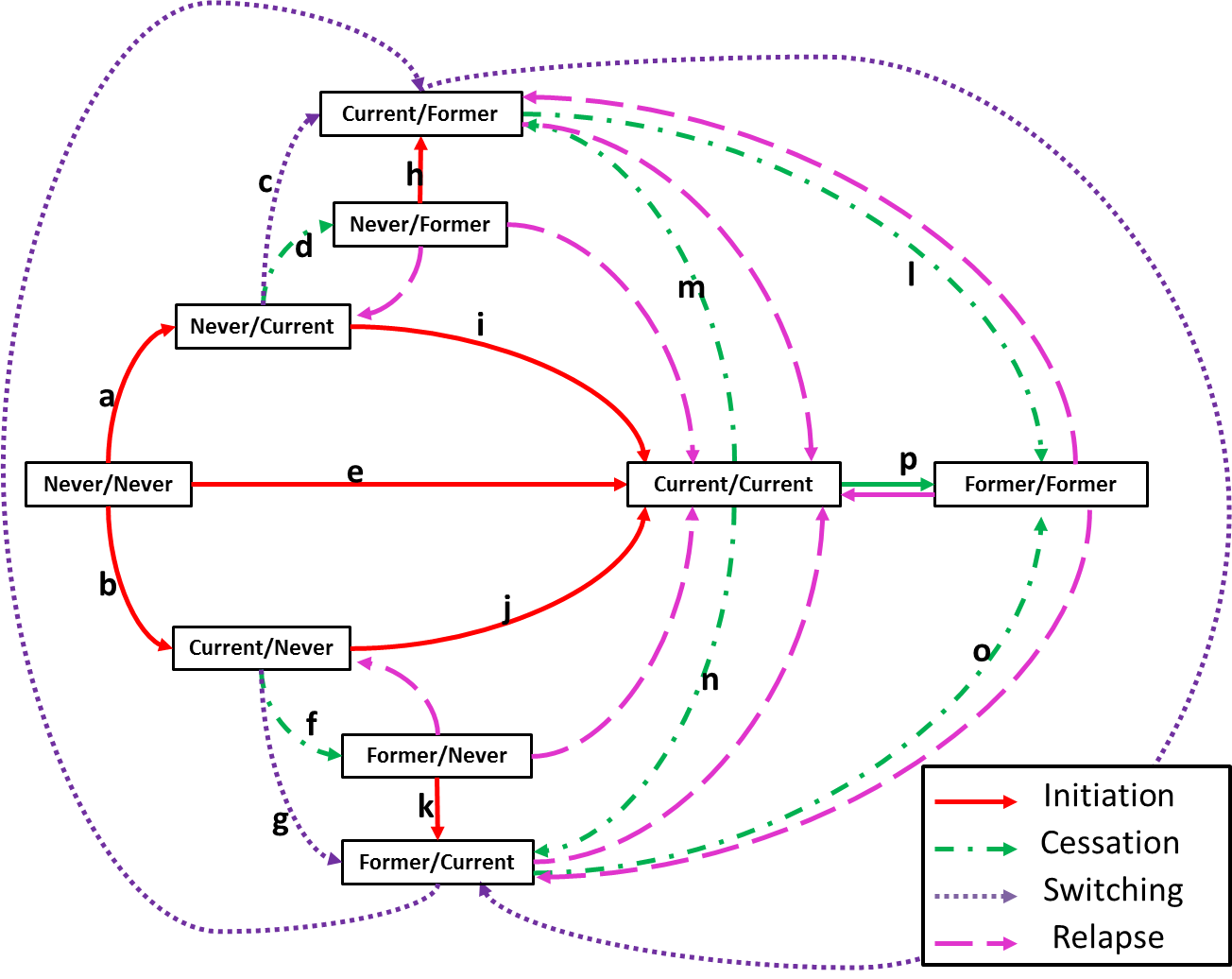


**Figure B: Tobacco-use-status transitions for a two-product model**

**Figure Note**: Transitions are categorized into four different behavior groups: initiation behaviors, cessation behaviors, switching behaviors, and relapse behaviors. The boxes represent the nine possible use statuses, with the first and second terms corresponding to the first and second tobacco products, respectively.

Deaths are calculated using all-cause mortality probabilities. Mortality probabilities vary by age, sex, and tobacco product use status. It is assumed that current and former tobacco product use may result in an elevated mortality risk, relative to never use of the products. Deaths decrease the overall size of the population (and subpopulations), and births contribute to the population size. Net international migration can increase or decrease the population size, depending on whether immigration or emigration is larger.

The model tracks the number of individuals in each subpopulation over time. At each time step[[1]](#footnote-1) and for each subpopulation, the model tracks the number of individuals within the subpopulation, the number of individuals that leave the subpopulation and join another subpopulation, and the number of individuals within the subpopulation who die. Key model output measures include prevalence of use for each tobacco product, mortality, and mortality attributable to tobacco product use.

# S1.2 Mathematical Model Description

Let $A=\left\{0,1,...,G,G^{+}\right\}$ be the set of ages, $S=\left\{male,female\right\}$ be the set of sexes, and *U* be the set of all possible tobacco product use status *N*-tuples considered in the model. Furthermore, let *F* denote the set of childbearing ages for females. Then Equations (1) through (9) provide a mathematical representation of the model, and Table A defines key model parameters and variables. Given that much of the data necessary to develop parameters is provided on an annual basis, time steps are generally taken to be one year increments, and parameters and variables are defined and described accordingly. A different sized time step could certainly be used, and interpretation of parameter and variable definition would need to change accordingly.

$Pop\left(0,s,u_{never},t_{i+1}\right)=b(s,t_{i+1})+m\left(0,s,u_{never},t_{i+1}\right)$ (1)

$Pop\left(0,s,u,t_{i+1}\right)=0,u\neq u_{never}$ (2)

$\begin{matrix} Pop\left(a+1,s,u,t_{i+1}\right)=\sum_{x\in U} Pop\left(a,s,x,t_{i}\right)\times p\left(x\to u\left|a,s,x,t_{i+1}\right.\right)\times\left[1-p\left(death\left|a,s,u,t_{i+1}\right.\right)\right] \\ \text{ }+m\left(a+1,s,u,t_{i+1}\right),\text{ }a=0,\text{ }...,G-1 \end{matrix}$ (3)

$\begin{matrix} Pop\left(G^{+},s,u,t_{i+1}\right)=\sum_{x\in U} Pop\left(G,s,x,t_{i}\right)\times p\left(x\to u\left|G,s,x,t_{i+1}\right.\right)\times\left[1-p\left(death\left|G,s,u,t_{i+1}\right.\right)\right] \\ +\sum_{x\in U} Pop\left(G^{+},s,x,t_{i}\right)\times p\left(x\to u\left|G^{+},s,x,t_{i+1}\right.\right)\times\left[1-p\left(death\left|G^{+},s,u,t_{i+1}\right.\right)\right] \\ +\text{ }m\left(G^{+},s,u,t_{i+1}\right) \end{matrix}$ (4)$m\left(a,s,u,t_{i+1}\right)=m_{frac}\left(a,s,u,t_{i+1}\right)\times m_{rate}\left(t_{i+1}\right)\times\sum_{a_{1}\in A} \sum_{s_{1}\in S} \sum_{u_{1}\in U} Pop\left(a_{1},s_{1},u_{1},t_{i}\right)$ (5)

$b\left(s,t_{i+1}\right)=b_{frac}\left(s,t_{i+1}\right)\times b_{rate}\left(t_{i+1}\right)\times\sum_{a\in F} \sum_{u\in U} \left[Pop\left(a,female,u,t_{i}\right)+m\left(a,female,u,t_{i+1}\right)\right]$ (6)

$p\left(death\left|a,s,u,t_{i}\right.\right)=RR\left(a,s,u,t_{i}\right)\times p\left(death\left|a,s,u_{never},t_{i}\right.\right)$ (7)

$prev\left(A_{1},S_{1},U_{1},t_{i}\right)=\frac{\sum_{a\in A_{1}} \sum_{s\in S_{1}} \sum_{u\in U_{1}} Pop\left(a,s,u,t_{i}\right)}{\sum_{a\in A_{1}} \sum_{s\in S_{1}} \sum_{u\in U} Pop\left(a,s,u,t_{i}\right)},\text{ }A_{1}\subset A,S_{1}\subset S,U_{1}\subset U$ (8)

$\begin{matrix} AD\left(A_{1},S_{1},t_{i+1}\right)=\sum_{a\in A_{1}} \sum_{s\in S_{1}} \sum_{x,u\in U} Pop\left(a,s,x,t_{i}\right)\times p\left(x\to u\left|a,s,x,t_{i+1}\right.\right)\times\left[RR\left(a,s,u,t_{i+1}\right)-1\right]\times p\left(death\left|a,s,u_{never},t_{i+1}\right.\right),\text{ } \\ \text{ }A_{1}\subset A,S_{1}\subset S \end{matrix}$ (9)

**Table A: Model parameters and variables**

| **Parameter** | **Description** | **Input Parameter or Output Variable** |
| --- | --- | --- |
| $Pop\left(a,s,u,t_{i}\right)$ | Number of individuals of age *a*, sex *s*, and tobacco use status *u* at year $t_{i}$. The population at year $t_{0}$is defined to be the initial population. | output (except for the initial population which is an input parameter) |
| $p\left(x\to u\left\vert a,s,x,t_{i+1}\right.\right)$ | Annual proportion of individuals of age *a*, sex *s*, and tobacco use status *x* that transition to tobacco use status *u* in the time interval $(t_{i},t_{i+1}]$. When *x = u*, this parameter represents the rate at which individuals maintain and do not change their tobacco use status. | input |
| $p\left(death\left\vert a,s,u,t_{i+1}\right.\right)$ | Annual proportion of individuals with age *a*, sex *s*, and tobacco use status *u* that die in the time interval $(t_{i},t_{i+1}]$ | input |
| $b_{rate}\left(t_{i+1}\right)$ | Annual number of births per female of childbearing age during the time interval $(t_{i},t_{i+1}]$ | input |
| $b_{frac}\left(s,t_{i+1}\right)$ | Fraction of births that are of sex *s* during the time interval $(t_{i},t_{i+1}]$ | input |
| $b(s,t_{i+1})$ | Number of births of sex *s* during the time interval $(t_{i},t_{i+1}]$ | output |
| $m_{rate}\left(t_{i+1}\right)$ | Annual number of net international migrants per person in the population entering/leaving during the time interval $(t_{i},t_{i+1}]$ | input |
| $m_{frac}\left(a,s,u,t_{i+1}\right)$ | Fraction of net international migrants entering/leaving the population during the time interval $(t_{i},t_{i+1}]$ of age *a*, sex *s*, and tobacco use status *u* | input |
| $m\left(a,s,u,t_{i+1}\right)$ | Number of net international migrants of age *a*, sex *s*, and tobacco use status *u* entering/leaving the population during the time interval $(t_{i},t_{i+1}]$ | output |
|  | Prevalence of individuals with tobacco use status in the subset $U_{1}\subset U$ among the population of age in the subset $A_{1}\subset A$ and sex in the subset $S_{1}\subset S$ at year $t_{i}$ | output |
| $AD\left(A_{1},S_{1},t_{i+1}\right)$ | Attributable deaths (from all products) among the population of age in the subset $A_{1}\subset A$* *and sex in the subset $S_{1}\subset S$ during the time interval $(t_{i},t_{i+1}]$ | output |

Equations [1] – [4] describe how subpopulations are tracked. The number of newborns (age 0) is determined solely by births and migration of infants less than age 1 (Equation [1]), and all newborns are assumed to have never used any of the tobacco products (Equation [2]). For older ages (Equations [3] and [4]), the size of a subpopulation is calculated by determining the number of people from the previous year who transition into a particular subpopulation and do not die and the number of net international migrants entering or leave the subpopulation.

Equations [5] and [6] calculate the number of net international migrants and births, respectively. Births are assumed to be linearly proportional to the number of females of childbearing ages in the population, and the number of immigrants is assumed to be linearly proportional to the entire population. These assumptions are made for consistency with U.S. Census Bureau projection methods. However, it should be noted that alternative birth and immigration functions could be easily substituted for the linear functions used in Equations [5] and [6].

The model assumes that the all-cause mortality proportion for a subpopulation is the product of the all-cause mortality relative risk (*RR*) for this subpopulation and the base mortality probability of dying for never users of any of the *N* tobacco products by sex and age (Equation [7]). (The base mortality probability for never users is specified in S2 Appendix). Because use of any combination of the *N* tobacco products is assumed to have no protective effect (relative to never use), all relative risks are greater than or equal to 1. Similar to births and migration, an alternative risk formulation could be substituted for this calculation.

Equations [8] and [9] describe how to calculate product use prevalence among a population and deaths attributable to use of any combination of the *N* tobacco products, respectively. The prevalence calculation is straightforward.

1. In its most general form, the model can use any specified time step for which appropriate parameters can be determined. In practice, annual time steps are most commonly used. [↑](#footnote-ref-1)
