# Supplementary material for: Modeling the Potential Effects of New Tobacco Products and Policies: A Dynamic Population Model for Multiple Product Use and Harm
Source: PLoS One. 2015 Mar 27;10(3):e0121008. doi: 10.1371/journal.pone.0121008 (PMC4376806; doi:10.1371/journal.pone.0121008)
Supplement: S2 Appendix — This appendix lists sources for population and cigarette smoking data that are used for the status quo model scenarios. The appendix also describes how input parameters were developed for the new product scenarios and relevant modeling assumptions. (DOCX) [file pone.0121008.s002.docx]

**S2 Appendix: Data Inputs and Assumptions**

# S2.1. Baseline One-Product Parameter Inputs and Assumptions

## S2.1.1 Baseline Scenario

The baseline one-product scenario projects the effect of tobacco use and harm in the US population over time where the single product is cigarette smoking and all parameters, except for the mortality scale factors, remain constant from 2000 through 2050. Fig. A shows the possible product use states and transitions involving the single product. Table A provides a summary of the one-product simulation parameter values that are used in our baseline scenario projections.


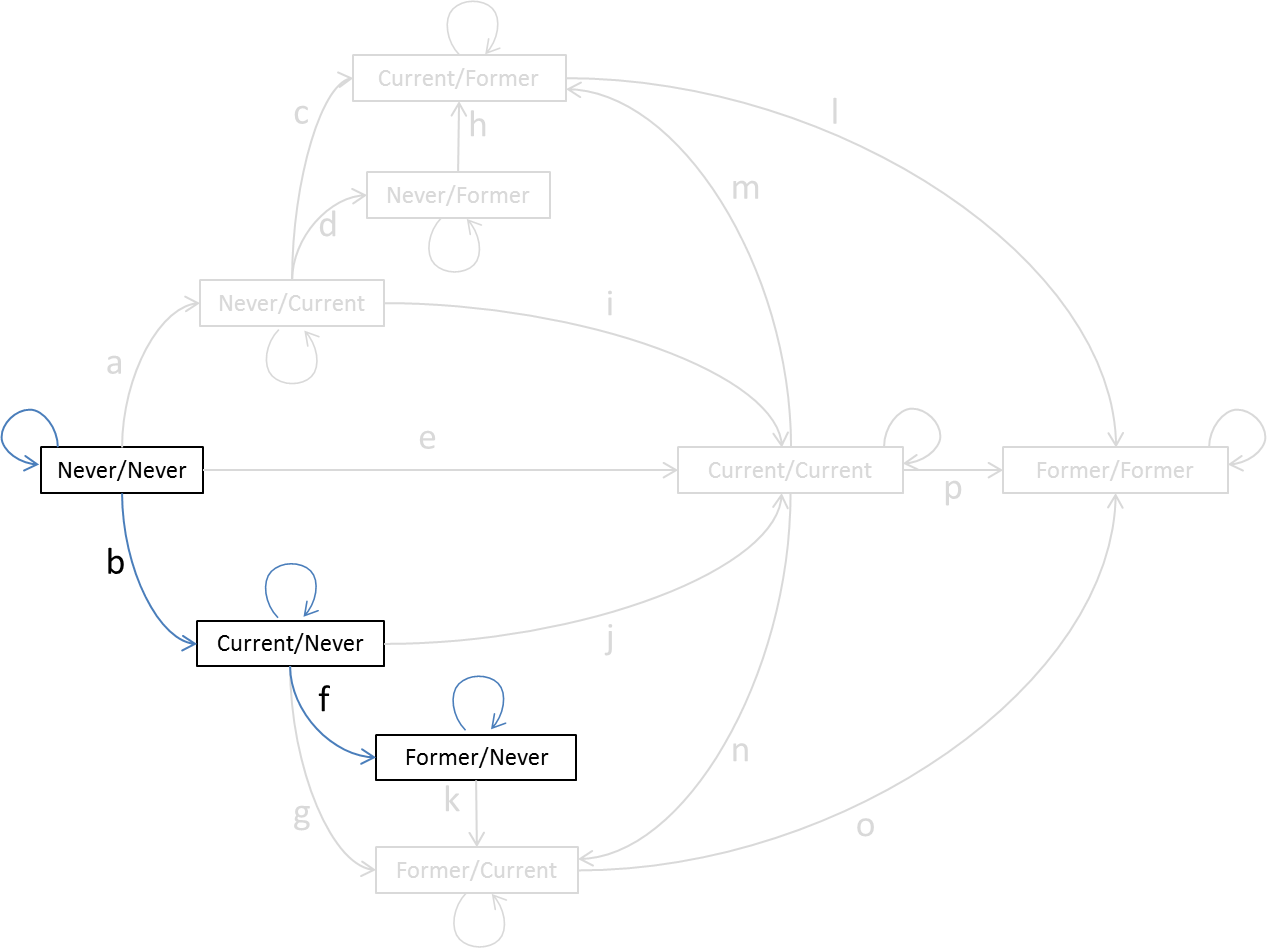


Figure A: One-product use states and transitions

Table A: Summary of baseline parameter inputs and data sources

| Model Component | Model Parameter | Data Source | Notes |
| --- | --- | --- | --- |
| Initial Population | Population distribution by sex and age | US Census National Population Estimates for 2000 [1]: <http://www.census.gov/population/projections/data/national/2008/downloadablefiles.html>, Table 1. |  |
|  | Cigarette smoking status (current, former, and never smoker) by sex, age, and time since cessation | National Center for Health Statistics (NCHS), National Health Interview Survey (NHIS) data from 2000 [2]. |  |
| Births | Birth rate by sex | Computed from US Census National Population Projections 2008 [1]: <http://www.census.gov/population/projections/data/national/2008/downloadablefiles.html>, Table 2. | Birth rates were calculated by dividing the projected number of births by sex by the projected number of females of ages 15-34 years for 2000-2050 from Census projections and applying these ratios to the number of females of ages 15-34 years projected for 2000-2050 by our model. |
| Net International Migration | Net migration rate by sex | Computed from US Census National Population Projections 2008 [1] - <http://www.census.gov/population/projections/data/national/2008/downloadablefiles.html>, Table 4. | Net migration rates by sex were calculated by dividing the projected number of net international migrants by the projected population size by sex for 2000-2050 from Census projections and applying these ratios to the population sizes by sex projected for 2000-2050 by our model. |
|  | Immigrant age distribution | US Census Bureau, The 2012 Statistical Abstract, the National Data Book, Table 40, 2012 [3]- <http://www.census.gov/compendia/statab/cats/population/native_and_foreign-born_populations.html>, Table 40. |  |
|  | Immigrant smoking prevalence by sex | NHIS data from 2007-2011 [2]. | Smoking prevalence was calculated for immigrants to the US of ages 18 years and over who had been in the US less than five years. |
| Deaths | Never-smoker death rate by sex and age  **Table A: Summary of baseline parameter inputs and data sources** (continued) | US Census Bureau, The 2012 Statistical Abstract, the National Data Book, Table 110, 2012 - death rates from 2000 for ages under 35 [3] and death rates calculated from National Health Interview Survey – Linked Mortality Files (NHIS-LMF) [4] data from 2002-2006 for ages 35 and over. | US death rates were used as death rates for never smokers of ages less than 35 years, given that smoking-attributable mortality is low at these ages. Never-smoker death rates for ages 35 years and over were estimated from NHIS-LMF data for NHIS Sample Adult Questionnaire participants from 1997-2004 followed for mortality through linkage with the National Death Index from 2002-2006. |
|  | Mortality adjustment factor by sex and age | US Vital Statistics and NHIS-LMF death rates from 2002-2006. US Vital Statistics death rates come from annual mortality reports.   - National Vital Statistics Report, Deaths: Final Data for 2002, Table 5 [5] <http://www.cdc.gov/nchs/data/nvsr/nvsr53/nvsr53_05acc.pdf> - National Vital Statistics Report, Deaths: Final Data for 2003, Table 5 [6] <http://www.cdc.gov/nchs/data/nvsr/nvsr54/nvsr54_13.pdf> - National Vital Statistics Report, Deaths: Final Data for 2004, Table 5 [7] <http://www.cdc.gov/nchs/data/nvsr/nvsr55/nvsr55_19.pdf> - National Vital Statistics Report, Deaths: Final Data for 2005, Table 5 [8] <http://www.cdc.gov/nchs/data/nvsr/nvsr56/nvsr56_10.pdf> - National Vital Statistics Report, Deaths: Final Data for 2006, Table 5 [9] <http://www.cdc.gov/nchs/data/nvsr/nvsr57/nvsr57_14.pdf> | NHIS-LMF never-smoker death rates were adjusted for low mortality in the NHIS’s civilian non-institutionalized population by multiplying the rates by the ratio of US Vital Statistics death rates divided by NHIS-LMF death rates by sex and age. |
|  | Mortality scaling factors | Calculated from U.S. death rates using Lee-Carter Method [10]. US death rates come from Human Mortality Database data. | Mortality scaling factors for 2000-2050 were calculated from US death rates for 1950-1999 using the Lee-Carter mortality projection method to account for expected age-specific changes in mortality over time. |
|  | Relative risk by sex, age, smoking status, and age at cessation for former smokers | Hazard ratios calculated from NHIS-LMF [4] data. | Hazard ratios were estimated for 1997-2004 NHIS Sample Adult Questionnaire participants followed for mortality through linkage with the National Death Index through the end of 2006. |
| Cigarette Smoking Transition Behaviors | Sex and age-specific initiation rates  **Table A: Summary of baseline parameter inputs and data sources** (continued) | Reconstructions of cohort smoking histories from NHIS data [11]. |  |
|  | Sex and age-specific cessation rates | Reconstructions of cohort smoking histories from NHIS data [11] . |  |
|  | Sex and age-specific relapse rates | Set to 0. |  |

## S2.1.2 Initial Population Estimates

The initial population in our model is the US population by sex, age, and smoking status for 2000, which is the initial year in our population projections.

### Year 2000 Population Distribution by Sex and Age

US population estimates for 2000 by sex and age come from US Census Bureau estimates [1] and are shown in Fig. B.

Figure B: US Population distribution by sex and age for 2000 from US Census Bureau estimates

### Year 2000 Smoking Prevalence by Sex, Age and Time since Cessation

US adult cigarette smoking prevalence for 2000 was estimated using National Health Interview Survey (NHIS) Sample Adult Questionnaire data [2]. NHIS is a national health survey of the US civilian non-institutionalized population that is conducted by the National Center for Health Statistics (NCHS) on an annual basis. NHIS data are used by the Centers for Disease Control and Prevention (CDC) to estimate smoking prevalence for the US adult population[12]. Prevalence was estimated for current and former smokers by sex and age, as shown in Table B. The proportional distribution of former smokers by time since cessation was also estimated, as shown in Tables C (Males) and D (Females). Smoking status was defined using the categories commonly used by the Centers for Disease Control and Prevention [12]. Current smokers reported having smoked at least 100 cigarettes in their lives and currently smoking every day or some days; former smokers reported having smoked at least 100 cigarettes in their lives and not currently smoking at all; and never smokers reported not having smoked at least 100 cigarettes in their lives. All NHIS estimates were calculated using SAS version 9.3 using the appropriate NHIS sample weights and taking into account the NHIS complex survey design in accordance with analytic guidelines provided by NCHS [24].

The NHIS Sample Child Questionnaire does not ask about tobacco use. Smoking prevalence for the US in 2000 for ages of less than 18 was obtained from estimates of cohort smoking histories for 1980-1984 birth cohorts that were reconstructed from cross-sectional NHIS data [11]. Current smoking prevalence by age for these cohorts was estimated by multiplying the estimated ever smoking prevalence at each age by the cumulative proportion of smokers who had not quit smoking by that age.

Figure C shows the initial population distribution in our model by sex, age, and smoking status which was generated using US Census Bureau population estimates and NHIS smoking prevalence data.

Figure C: US population distribution by sex, age, and smoking status for 2000 from US Census Bureau estimates and the National Health Interview Survey

Table B: Adult smoking prevalence by sex and age, National Health Interview Survey, 2000

| Age | Males | | | |  | Females | | | |
| --- | --- | --- | --- | --- | --- | --- | --- | --- | --- |
|  | **Current** | **95% CI** | **Former** | **95% CI** |  | **Current** | **95% CI** | **Former** | **95% CI** |
| 18-24 | 28.5% | 25.9%, 31.1% | 7.4% | 5.9%, 9.0% |  | 25.1% | 22.6%, 27.5% | 8.0% | 6.6%, 9.4% |
| 25-34 | 29.0% | 27.0%, 31.1% | 12.3% | 10.9%, 13.7% |  | 22.5% | 20.9%, 24.1% | 10.9% | 9.6%, 12.2% |
| 35-44 | 30.2% | 28.2%, 32.2% | 16.5% | 15.0%, 17.9% |  | 26.2% | 24.6%, 27.7% | 17.1% | 15.6%, 18.5% |
| 45-54 | 28.8% | 26.8%, 30.9% | 28.9% | 26.6%, 31.2% |  | 22.2% | 20.5%, 23.8% | 23.1% | 21.3%, 24.9% |
| 55-64 | 22.6% | 20.4%, 24.8% | 45.0% | 42.3%, 47.7% |  | 20.8% | 18.9%, 22.8% | 28.3% | 26.1%, 30.5% |
| 65-74 | 13.7% | 11.7%, 15.6% | 53.9% | 50.9%, 56.9% |  | 12.2% | 10.5%, 13.8% | 28.8% | 26.3%, 31.3% |
| 75-84 | 5.7% | 4.0%, 7.3% | 58.7% | 54.8%, 62.6% |  | 6.9% | 5.6%, 8.2% | 25.1% | 22.6%, 27.6% |
| 85+ | 3.8% | 1.0%, 6.6% | 58.3% | 49.8%, 66.7% |  | 3.8% | 2.1%, 5.6% | 19.3% | 14.5%, 23.7% |
| Total | 25.7% | 24.8%, 26.6% | 25.8% | 24.9%, 26.7% |  | 21.0% | 20.2%, 21.8% | 18.9% | 18.2%, 19.6% |

Table C: Proportional distribution of male former smokers by time since cessation by sex and age, National Health Interview Survey, 2000

| Age | Years since Cessation | | | | | | | | | | | | | | |
| --- | --- | --- | --- | --- | --- | --- | --- | --- | --- | --- | --- | --- | --- | --- | --- |
|  | **0-4** | **5-9** | **10-14** | **15-19** | **20-24** | **25-29** | **30-34** | **35-39** | **40-44** | **45-49** | **50-54** | **55-59** | **60-64** | **65-69** | **70+** |
| 18-24 | 88.6% | 11.4% | - | - | - | - | - | - | - | - | - | - | - | - | - |
| 25-34 | 58.7% | 24.0% | 15.0% | 2.3% | - | - | - | - | - | - | - | - | - | - | - |
| 35-44 | 31.9% | 18.1% | 19.9% | 13.8% | 14.2% | 2.1% | - | - | - | - | - | - | - | - | - |
| 45-54 | 18.5% | 13.4% | 16.4% | 15.1% | 16.2% | 11.5% | 7.9% | 1.0% | - | - | - | - | - | - | - |
| 55-64 | 16.6% | 10.5% | 14.5% | 14.3% | 13.5% | 12.1% | 8.9% | 5.0% | 4.4% | 0.1% | - | - | - | - | - |
| 65-74 | 9.7% | 8.3% | 11.0% | 7.9% | 13.9% | 8.2% | 15.4% | 7.9% | 10.9% | 4.7% | 1.9% | 0.2% | - | - | - |
| 75-84 | 6.4% | 7.3% | 8.5% | 9.5% | 14.1% | 9.6% | 12.8% | 6.3% | 12.9% | 4.2% | 6.2% | 0.2% | 1.5% | 0.2% | - |
| 85+ | 6.6% | 7.0% | 8.2% | 8.2% | 10.4% | 11.1% | 11.5% | 5.8% | 13.6% | 2.5% | 2.4% | 4.5% | 3.1% | 1.6% | 3.5% |

**Table D: Proportional distribution of female former smokers by time since cessation by sex and age, National Health Interview Survey, 2000**

| Age | Years since Cessation | | | | | | | | | | | | | | |
| --- | --- | --- | --- | --- | --- | --- | --- | --- | --- | --- | --- | --- | --- | --- | --- |
|  | **0-4** | **5-9** | **10-14** | **15-19** | **20-24** | **25-29** | **30-34** | **35-39** | **40-44** | **45-49** | **50-54** | **55-59** | **60-64** | **65-69** | **70+** |
| 18-24 | 92.6% | 7.4% | - | - | - | - | - | - | - | - | - | - | - | - | - |
| 25-34 | 60.1% | 24.6% | 13.6% | 1.7% | - | - | - | - | - | - | - | - | - | - | - |
| 35-44 | 27.8% | 18.2% | 21.9% | 16.4% | 13.4% | 2.3% | - | - | - | - | - | - | - | - | - |
| 45-54 | 22.6% | 11.5% | 13.5% | 18.0% | 15.3% | 10.9% | 8.0% | 0.3% | - | - | - | - | - | - | - |
| 55-64 | 19.0% | 9.9% | 13.2% | 13.7% | 13.4% | 9.4% | 11.5% | 5.7% | 3.4% | 0.8% | - | - | - | - | - |
| 65-74 | 13.1% | 14.0% | 13.5% | 11.6% | 11.1% | 9.3% | 10.9% | 5.0% | 6.0% | 1.9% | 3.5% | - | - | - | - |
| 75-84 | 9.5% | 8.8% | 11.3% | 10.8% | 15.0% | 8.5% | 11.6% | 6.4% | 7.6% | 2.8% | 4.1% | 1.6% | 1.9% | - | - |
| 85+ | 14.4% | 3.4% | 16.5% | 4.9% | 11.6% | 4.6% | 11.7% | 3.0% | 4.0% | 6.7% | 7.5% | 4.0% | 5.0% | 2.7% | - |

## S2.1.3 Births and Net International Migration

The US Census Bureau provides birth, net international migration, and population projections for the US population from 2001-2050 [1]. We use these projections to parameterize our model with birth and net migration rates. We limit fertility to ages less than 35 years to prevent any effects of tobacco-attributable mortality on fertility in different scenarios, given that our model introduces tobacco-attributable mortality at age 35. We compute an annual birth rate ($BR$) by sex of infant ($s$) for the time interval *t* to *t* + 1 for use in the model by dividing the projected birth count by the projected female population count for ages 15-34 years for each year from 2001-2050.

$$\begin{matrix} B_{t,t+1}(s) & = & \text{Census projected births of sex=}s\text{ in the interval} t \text{to} t+1 \\ \sum_{a=15}^{34} P_{t}(\text{female},a) & = & \text{Census projected female population ages 15-34 at time }t \\ {BR}_{t,t+1}(s) & = & \frac{B_{t,t+1}(s)}{\sum_{a=15}^{34} P_{t}(\text{female},a)} \end{matrix}$$

Birth rates used in the model for the period from 2001-2050 range between 5.2 and 5.4 male births and 5.0 and 5.1 female births per 100 women. We then use the calculated birth rates to project the number of births by sex in the interval *t* to *t* + 1 by applying the rate to the projected female population ages 15-34 years in our model at time *t*, where $u$ tracks the tobacco use state.

$$\begin{matrix} \sum_{a=15}^{34} \sum_{u} P_{t}(\text{female},a,u) & = & \text{model projected female population ages of 15-34 at time} t \\ B_{t,t+1}(s,\text{never}) & = & {BR}_{t,t+1}(s)\cdot\sum_{a=15}^{34} \sum_{u} P_{t}\left( \text{female},a,u \right) \end{matrix}$$

The Census Bureau also provides net international migration and total population projections by year from 2001-2050 [1]. We compute a net migration rate ($MR$) by sex ($s$) for the interval *t* to *t* + 1 for use in our model by dividing the net international migration projections by the total population projections by year.

$$\begin{matrix} \sum_{a} M_{t,t+1}(s,a) & = & \text{Census projected net migrants with sex=}s\text{ for the interval} t \text{to} t+1 \\ \sum_{s,a} P_{t}(s,a) & = & \text{Census projected total population at time} t \\ {MR}_{t,t+1}(s) & = & \frac{\sum_{a} M_{t,t+1}(s,a)}{\sum_{s,a} P_{t}(s,a)} \end{matrix}$$

Projected net migration for the US from 2001-2050 is consistently positive, indicating greater immigration than emigration. Calculated net international migration rates used in our model are between 1.2 and 2.2 per 1000 for males and between 1.5 and 2.5 per 1000 for females.

We use these calculated net migration rates in our model to project the net number of immigrants by sex ($s$) in the interval *t* to *t* + 1 by applying them to the projected total population in the model at time *t*.

$$\begin{matrix} \sum_{s,a,u} P_{t}(s,a,u) & = & \text{model projected total population at time} t \\ M_{t,t+1}(s) & = & {MR}_{t,t+1}\left( s \right)\cdot\sum_{s,a,u} P_{t}\left( s,a,u \right) \end{matrix}$$

We obtained the age distribution of immigrants, $M_{t,t+1}(s,a)$, from US Census Bureau estimates of the age distribution of immigrants in the US in 2010 who had arrived in the US between 2000 and 2010 [3].

Smoking prevalence for the incoming net international migrant population, $M_{t,t+1}(s,a,u)$, is based on an analysis of 2007-2011 NHIS data for adult immigrants who had been in the US less than five years. These years were the most recent with NHIS data available, and smoking prevalence was estimated from five years of data for immigrants who had been in the US for less than five years to increase the precision of the estimates. The current smoking prevalence for these immigrants was 19.1% for males and 4.4% for females and the former smoking prevalence was 13.1% for males and 5.6% for females (NHIS). These estimates are used in the model for incoming immigrants ages 18 years and over throughout the projection period from 2001-2050. We assigned years since cessation to the former smoker immigrant population using the distributions by sex and age observed for the US in 2000 NHIS data, as described above.

## S2.1.4 Deaths

### S2.1.4.1 Death Rates for Never Smokers

Never-smoker death rates provide the base for estimates of mortality and smoking-attributable mortality in our model. These rates are projected for the period from 2000-2050. US death rates for the year 2000 by sex and age from vital statistics data are used to project never-smoker death rates for ages of less than 35 years [13], given that smoking-attributable mortality is low at these ages. Never-smoker death rates for ages of 35 years and over are projected from estimates from National Health Interview Survey – Linked Mortality Files (NHIS-LMF) data [4]. NHIS-LMF data provide recent, nationally representative estimates of mortality risks and have been used extensively in the analysis of mortality risks by smoking status [14,15] and in modeling smoking-attributable mortality [16]. The NHIS-LMF data used in our analysis come from NHIS Sample Adult Questionnaire participants from 1997-2004 followed for mortality through linkage with the National Death Index through the end of 2006. The NHIS-LMF never-smoker death rates ($m)$ are estimated by ten-year age group as the ratio of deaths ($d)$ to person-time ($L$) for never smokers ($u=ns$) during mortality follow-up from 2002-2006.

$$NHIS-LMF m(s,a,ns)=\frac{d(s,a,ns)}{L (s,a, ns)}$$

The NHIS-LMF never-smoker death rates are shown in Table E. NHIS-LMF death rates tend to be somewhat lower than US death rates, given that the NHIS considers the US civilian non-institutionalized population, which excludes individuals in long-term care facilities and nursing homes. The NHIS-LMF never-smoker death rates were therefore adjusted by multiplying them by the ratio of US death rates from vital statistics data divided by NHIS-LMF death rates by sex and age for 2002-2006.

$${NHIS-LMF}_{adj} m(s,a, ns) = NHIS-LMF m(s,a, ns)\cdot\frac{US m(s,a)}{NHIS-LMF m(s,a)}$$

These ratios are shown in Table F (Males) and Table G (Females).

Table E: Never-smoker death rates per 100,000 person-years from mortality follow-up from 2002-2006 for 1997-2004 NHIS Sample Adult Questionnaire participants

| Age | Males | |  | Females | |
| --- | --- | --- | --- | --- | --- |
|  | **Death Rate per 100,000 person years** | **Standard Error** |  | **Death Rate per 100,000 person years** | **Standard Error** |
| 35-44 | 143 | 18 |  | 83 | 12 |
| 45-54 | 361 | 30 |  | 201 | 21 |
| 55-64 | 564 | 54 |  | 489 | 40 |
| 65-74 | 1,731 | 127 |  | 1,055 | 58 |
| 75-84 | 4,367 | 255 |  | 3,198 | 112 |
| 85+ | 14,151 | 707 |  | 10,498 | 267 |

Table F: Male death rates from follow-up from 2002-2006 for 1997-2004 NHIS Sample Adult Questionnaire participants and mean 2002-2006 US death rates by sex and age

| Age | Death Rates | | | Ratio |
| --- | --- | --- | --- | --- |
|  | **NHIS-LMF Rate** | **Standard Error** | **US Rate** |  |
| 35-44 | 195 | 16 | 248 | 1.268 |
| 45-54 | 530 | 25 | 547 | 1.031 |
| 55-64 | 1,010 | 44 | 1,144 | 1.133 |
| 65-74 | 2,665 | 92 | 2,680 | 1.005 |
| 75-84 | 5,918 | 156 | 6,465 | 1.092 |
| 85+ | 15,323 | 483 | 15,256 | 0.996 |

Table G: Female death rates from follow-up from 2002-2006 for 1997-2004 NHIS Sample Adult Questionnaire participants and mean 2002-2006 US death rates by sex and age

| Age | Death Rates | | | Ratio |
| --- | --- | --- | --- | --- |
|  | **NHIS-LMF Rate** | **Standard Error** | **US Rate** |  |
| 35-44 | 122 | 12 | 146 | 1.192 |
| 45-54 | 278 | 17 | 317 | 1.141 |
| 55-64 | 673 | 30 | 713 | 1.060 |
| 65-74 | 1,540 | 57 | 1,773 | 1.151 |
| 75-84 | 4,027 | 103 | 4,573 | 1.135 |
| 85+ | 11,075 | 259 | 13,522 | 1.221 |

### S2.1.4.2 Mortality Projections

Our model projects never-smoker death rates from 2000-2050. Rates are projected using mortality scaling factors obtained from the Lee-Carter mortality forecasting method [10] as implemented in the demography package version 1.16 for R [17]. This method was used to project expected age-specific changes in mortality over time based on observed death rates. The Lee-Carter models death rates as a function of age ($x$) and time ($t$) as:

$$\ln\left( m_{x,t} \right)=a_{x}+b_{x}k_{t}+e_{x,t}$$

where $a_{x}$ is an age coefficient that defines the basic mortality level by age, $b_{x}$ is an age coefficient that identifies change in mortality over time, $k_{t}$ is a parameter for mortality change over time, and $e_{x,t}$ is an error term. The model is fit to data using a least-squares solution found from the singular value decomposition. Death rates are then forecast by modeling $k_{t}$ as a random walk with drift:

$$k_{t}=c+k_{t-1}+u_{t}$$

where $c$ is a drift term representing the average annual change in $k_{t}$ and $u_{t}$ is an error term.

The Lee-Carter method was used to project US death rates from 2000-2050 by sex and age using observed US death rates from 1950-1999. The resulting projected rates were centered around 2000 for ages of less than 35 years and around 2004 for ages of 35 years and over. Mortality scaling factors by sex and age were calculated as the ratio of projected rates for 2000-2050 to the projected rates for 2000 for ages of less than 35 years and for 2004 for ages of 35 years and over. These scaling factors were then applied to the never-smoker death rates explained above for 2000 for ages of less than 35 years and for 2004 for ages of 35 years and over to produce projected never-smoker death rates by sex and age for 2000-2050.

$$msf\left( s, a<35, t=2000-2050 \right)=\frac{projected US m(s,a,t)}{projected US m(s,a,t=2000)}$$

$$msf\left( s, a\geq35, t=2000-2050 \right)=\frac{projected US m(s,a,t)}{projected US m(s,a,t=2004)}$$

$${NHIS-LMF}_{adj} m\left( s,a, ns, t \right)={NHIS-LMF}_{adj} m\left( s,a, ns \right)\cdot msf(s,a,t)$$

The scaling factors by ten-year intervals are shown in Table H.

Table H: Mortality scaling factors obtained from Lee-Carter mortality forecasting method for US from 2000-2050 by sex and age

| Age | Males | | | | | |  | Females | | | | | |
| --- | --- | --- | --- | --- | --- | --- | --- | --- | --- | --- | --- | --- | --- |
|  | **2000** | **2010** | **2020** | **2030** | **2040** | **2050** |  | **2000** | **2010** | **2020** | **2030** | **2040** | **2050** |
| 0 | 1 | 0.719 | 0.516 | 0.371 | 0.267 | 0.192 |  | 1 | 0.752 | 0.565 | 0.425 | 0.320 | 0.240 |
| 1 | 1 | 0.769 | 0.592 | 0.455 | 0.350 | 0.269 |  | 1 | 0.779 | 0.607 | 0.473 | 0.369 | 0.288 |
| 5 | 1 | 0.774 | 0.599 | 0.463 | 0.359 | 0.278 |  | 1 | 0.800 | 0.640 | 0.512 | 0.409 | 0.327 |
| 10 | 1 | 0.830 | 0.689 | 0.572 | 0.475 | 0.394 |  | 1 | 0.851 | 0.725 | 0.617 | 0.525 | 0.447 |
| 15 | 1 | 0.942 | 0.887 | 0.835 | 0.786 | 0.740 |  | 1 | 0.921 | 0.848 | 0.781 | 0.719 | 0.662 |
| 20 | 1 | 0.934 | 0.872 | 0.814 | 0.760 | 0.709 |  | 1 | 0.895 | 0.800 | 0.716 | 0.641 | 0.573 |
| 25 | 1 | 0.957 | 0.917 | 0.877 | 0.840 | 0.804 |  | 1 | 0.886 | 0.785 | 0.695 | 0.616 | 0.545 |
| 30 | 1 | 0.972 | 0.944 | 0.917 | 0.891 | 0.866 |  | 1 | 0.878 | 0.771 | 0.677 | 0.595 | 0.522 |
| 35 | 1.024 | 0.966 | 0.911 | 0.859 | 0.810 | 0.764 |  | 1.057 | 0.920 | 0.801 | 0.697 | 0.606 | 0.528 |
| 40 | 1.043 | 0.939 | 0.845 | 0.761 | 0.685 | 0.617 |  | 1.060 | 0.916 | 0.791 | 0.683 | 0.590 | 0.509 |
| 45 | 1.056 | 0.921 | 0.803 | 0.701 | 0.611 | 0.533 |  | 1.057 | 0.921 | 0.802 | 0.699 | 0.609 | 0.530 |
| 50 | 1.064 | 0.911 | 0.780 | 0.668 | 0.572 | 0.490 |  | 1.051 | 0.928 | 0.819 | 0.722 | 0.637 | 0.562 |
| 55 | 1.058 | 0.919 | 0.797 | 0.692 | 0.601 | 0.521 |  | 1.041 | 0.942 | 0.852 | 0.771 | 0.698 | 0.631 |
| 60 | 1.053 | 0.925 | 0.813 | 0.714 | 0.627 | 0.551 |  | 1.039 | 0.945 | 0.860 | 0.782 | 0.712 | 0.648 |
| 65 | 1.048 | 0.933 | 0.831 | 0.740 | 0.659 | 0.586 |  | 1.041 | 0.942 | 0.853 | 0.772 | 0.699 | 0.633 |
| 70 | 1.039 | 0.944 | 0.857 | 0.778 | 0.706 | 0.641 |  | 1.044 | 0.937 | 0.841 | 0.754 | 0.677 | 0.607 |
| 75 | 1.037 | 0.947 | 0.865 | 0.790 | 0.722 | 0.660 |  | 1.052 | 0.927 | 0.818 | 0.721 | 0.636 | 0.561 |
| 80 | 1.029 | 0.958 | 0.893 | 0.832 | 0.775 | 0.722 |  | 1.045 | 0.936 | 0.839 | 0.752 | 0.673 | 0.603 |
| 85 | 1.017 | 0.976 | 0.936 | 0.899 | 0.863 | 0.828 |  | 1.025 | 0.964 | 0.906 | 0.853 | 0.802 | 0.754 |

### S2.1.4.3 Mortality Probability Conversion and Calculations

Age-specific death rates by sex and time were assumed to be equal to these adjusted and projected death rates by sex, time, and ten-year age group. These age-specific death rates ($m$) which have person-time as their denominator, were then converted to age-specific probabilities of dying ($q$), which have persons-at-risk as their denominator, for use in calculating mortality in the population model. The conversion was made using the standard demographic formula [18]:

$$q_{x}= \frac{m_{x}}{1+(1-a_{x})m_{x}}$$

where $x$ is year of age and $a_{x}$ is the average length of time lived at that age by people who die at that age. Values for $a_{x}$ at young ages were taken from Coale and Demeny model life tables and were 0.045+2.684*$m_{0}$ for males and 0.053+2.800*$m_{0}$ for females at age 0 and (1.651-2.816*$m_{0}$)/4 for males and (1.522-1.518*$m_{0}$)/4 for females at ages of 1-4 years [18]. Values for $a_{x}$ at ages of 5 years and over were set equal to 0.5.

Never-smoker probabilities of dying were then multiplied by relative risks ($RR$ ) for current and former smokers compared to never smokers by sex and age to produce probabilities of dying for current and former smokers for use in the population model.

$$q\left( s,a, u, t \right)={NHIS-LMF}_{adj} q\left( s,a, ns,t \right)\cdot RR(s,a,u, t)$$

### S2.1.4.4 Smoking Relative Risks

Relative risks by smoking status were estimated as hazard ratios using NHIS-LMF data for 1997-2004 NHIS Sample Adult Questionnaire data with mortality follow-up through the end of 2006. The hazard ratios compare mortality risks for current and former smokers to those of never smokers; this comparison is shown in Table I (current smokers), Table J (male former smokers observed), Table K (female former smokers observed), and Table L (former smokers used in modeling). The hazard ratios were estimated by sex and age using age as the time scale and adjusting for race/ethnicity, educational attainment, alcohol consumption, and body mass index. Hazard ratios for former smokers were also estimated by age at cessation. Estimated hazard ratios for the most recent quitters among former smokers were often quite high, sometimes higher than estimated hazard ratios for current smokers of the same age. Similar results have been observed previously for recent quitters [19,20], and the most common explanation is that some of these individuals quit smoking due to symptoms or diagnoses of smoking-related illnesses but still had increased mortality risks after smoking cessation. To prevent increases in mortality from occurring in scenarios with increased smoking cessation, we set the maximum hazard ratio for former smokers equal to the hazard ratio for current smokers of the same age and sex. Estimated hazard ratios for former smokers who quit before the age of 40 were generally slightly above or below 1.0, consistent with previous research that has found limited increased mortality risks for former smokers who stopped smoking before this age [14]. We therefore set these hazard ratios to 1.0 for use in the population model.

Table I: Current-smoker mortality hazard ratios from follow-up from 1997-2006 for 1997-2004 NHIS Sample Adult Questionnaire participants by sex, age, and smoking status

| Age | Males | | Females | |
| --- | --- | --- | --- | --- |
|  | **HR** | **95% CI** | **HR** | **95% CI** |
| 35-44 | 1.84 | (1.34, 2.52) | 3.34 | (2.37, 4.72) |
| 45-54 | 2.04 | (1.62, 2.58) | 2.42 | (1.87, 3.12) |
| 55-64 | 2.79 | (2.28, 3.40) | 2.35 | (1.92, 2.87) |
| 65-74 | 2.77 | (2.34, 3.29) | 2.90 | (2.48, 3.39) |
| 75-84 | 2.19 | (1.88, 2.54) | 2.57 | (2.28, 2.90) |
| 85+ | 1.36 | (1.01, 1.84) | 1.56 | (1.28, 1.90) |

Table J: Male former smoker observed mortality hazard ratios from follow-up from 1997-2006 for 1997-2004 NHIS Sample Adult Questionnaire participants by sex, age, and smoking status

| Age | Age at Cessation | | | | | | | |
| --- | --- | --- | --- | --- | --- | --- | --- | --- |
|  | **< 40** | | **40-49** | | **50-59** | | **60+** | |
|  | **HR** | **95% CI** | **HR** | **95% CI** | **HR** | **95% CI** | **HR** | **95% CI** |
| 35-44 | 1.05 | (0.67, 1.64) | 5.78 | (2.01, 16.59) |  |  |  |  |
| 45-54 | 0.99 | (0.73, 1.35) | 1.68 | (1.18, 2.39) | 4.60 | (2.39, 8.86) |  |  |
| 55-64 | 0.96 | (0.71, 1.28) | 1.54 | (1.16, 2.06) | 2.44 | (1.77, 3.35) | 2.56 | (1.22, 5.39) |
| 65-74 | 0.76 | (0.60, 0.95) | 1.19 | (0.96, 1.48) | 1.87 | (1.52, 2.30) | 2.20 | (1.82, 2.67) |
| 75-84 | 1.02 | (0.87, 1.21) | 1.43 | (1.20, 1.69) | 1.50 | (1.29, 1.74) | 1.99 | (1.75, 2.77) |
| 85+ | 1.07 | (0.89, 1.29) | 1.12 | (0.93, 1.36) | 1.04 | (0.86, 1.26) | 1.35 | (1.15, 1.58) |

Table K: Female former smoker observed mortality hazard ratios from follow-up from 1997-2006 for 1997-2004 NHIS Sample Adult Questionnaire participants by sex, age, and smoking status

| Age | Age at Cessation | | | | | | | |
| --- | --- | --- | --- | --- | --- | --- | --- | --- |
|  | **< 40** | | **40-49** | | **50-59** | | **60+** | |
|  | **HR** | **95% CI** | **HR** | **95% CI** | **HR** | **95% CI** | **HR** | **95% CI** |
| 35-44 | 1.67 | (1.00, 2.81) | 3.70 | (0.69, 19.97) |  |  |  |  |
| 45-54 | 1.05 | (0.70, 1.57) | 1.57 | (0.96, 2.56) | 6.06 | (2.08, 17.59) |  |  |
| 55-64 | 0.81 | (0.52, 1.25) | 1.71 | (1.18, 2.47) | 2.56 | (1.92, 3.41) | 6.30 | (3.39, 11.73) |
| 65-74 | 1.06 | (0.76, 1.47) | 1.56 | (1.19, 2.05) | 1.94 | (1.58, 2.38) | 3.20 | (2.54, 4.03) |
| 75-84 | 1.09 | (0.85, 1.39) | 1.38 | (1.11, 1.70) | 1.52 | (1.26, 1.83) | 2.23 | (1.99, 2.50) |
| 85+ | 1.02 | (0.79, 1.31) | 1.09 | (0.82, 1.44) | 1.37 | (1.11, 1.70) | 1.54 | (1.35, 1.75) |

Table L: Former-smoker mortality hazard ratios used in modeling

| Age | Males | | | |  | Females | | | |
| --- | --- | --- | --- | --- | --- | --- | --- | --- | --- |
|  | Age at Cessation | | | |  | Age at Cessation | | | |
|  | **< 40** | **40-49** | **50-59** | **60+** |  | **< 40** | **40-49** | **50-59** | **60+** |
| 35-44 | 1.00 | 1.84 |  |  |  | 1.00 | 3.34 |  |  |
| 45-54 | 1.00 | 1.68 | 2.04 |  |  | 1.00 | 1.57 | 2.42 |  |
| 55-64 | 1.00 | 1.54 | 2.44 | 2.56 |  | 1.00 | 1.71 | 2.35 | 2.35 |
| 65-74 | 1.00 | 1.19 | 1.87 | 2.20 |  | 1.00 | 1.56 | 1.94 | 2.90 |
| 75-84 | 1.00 | 1.43 | 1.50 | 1.99 |  | 1.00 | 1.38 | 1.52 | 2.23 |
| 85+ | 1.00 | 1.00 | 1.00 | 1.35 |  | 1.00 | 1.00 | 1.37 | 1.54 |

## S2.1.5 Smoking Initiation and Cessation

We use sex- and age-specific cigarette initiation and cessation rates in our model that were produced from US birth cohort smoking history data. The estimates were generated as part of the CISNET (Cancer Intervention and Surveillance Modeling Network) Lung Working Group, which is sponsored by the National Cancer Institute, and have been used to model and quantify the effect of reduced cigarette smoking on lung cancer mortality in the US from 1975 to 2000 [21]. Working group researchers estimated cohort smoking histories using 25 NHIS surveys administered from 1965 to 2001 [11]. The researchers estimated current- and ever-smoking prevalence and initiation and cessation rates by age and sex for five-year birth cohorts for individuals born between 1900 and 1984. A detailed explanation of their methodological approach and the resulting data are available on-line [22].

We use cohort age-specific smoking initiation rates for the period closest to 2000, the initial year in our population model projections. We use initiation rates for ages 0-19 years from 1980-1984 birth cohorts, rates for ages 20-24 from 1975-1979 cohorts, rates for ages 25-29 from 1970-1974 cohorts, and rates for age 30 from 1965-1969 cohorts. We do not include cigarette initiation beyond the age of 30 in our model, given that the overwhelming majority of smoking initiation has occurred by this age [23]. Figure D shows the estimated initiation rates by sex and age that are used in the model. The rates reflect initiation to established use as indicated by NHIS participants reporting if they had smoked at least 100 cigarettes in their lives. These age-specific initiation rates are assumed to remain constant throughout the projection period in the baseline scenario.

We also use smoking cessation rates in the model that come from CISNET cohort data. Rates for ages 1-18 years come from 1980-1984 birth cohorts, rates for ages 19-24 come from 1975-1979 cohorts, rates for ages 25-29 come from 1970-1974 cohorts, and so on through ages 83-85 from 1900-1904 cohorts. Cessation rates are available from the cohort data through age 85 for males and age 84 for females. Beyond these ages, cessation rates in the model are assumed to remain equal to the last available estimate from the cohort data. Figure E shows the estimated cessation rates by sex and age that are used in the model. The model allows for relapse, but transition probabilities for relapse behaviors are set to zero in the modeling simulations presented here because the CISNET cessation rates reflect successful smoking cessation for at least two years. These age-specific cessation rates are assumed to remain constant throughout the projection period in the baseline scenario.

**Figure D: Cigarette Smoking Initiation Rates by Sex and Age from CISNET Cohort Smoking Histories**

**Figure E: Cigarette Smoking Cessation Rates by Sex and Age from CISNET Cohort Smoking Histories**

# S2.2. Alternative Two-Product Scenario Parameter Inputs and Assumptions

## S2.2.1 Two-Product Scenarios

The alternative two-product scenarios project the effect of introduction of a hypothetical new tobacco product on tobacco use and harm in the US population over time. The scenario begins in 2000 with cigarettes as the only product, using the input values described in Section S2.1. The hypothetical new product is introduced in 2003. Figure F shows the possible product use states and transitions involving the two products. Table M provides a summary of the two-product simulation parameter values that are allowed to vary in the model projections and their implementation in the model. Sex- and age-specific initiation rates and excess relative risk for the new product are defined as proportions of initiation rates and excess relative risk for cigarettes. Initiation for the hypothetical new tobacco product and switching from this new product to cigarettes is not allowed after age 30, given that most tobacco initiation occurs among young people. Initiation rates for a product for former users of the other product (transitions **h** and **k** in Figure F) are set equal to initiation rates for this product for never users of the other product. Initiation to dual use in a single year is assumed to be minimal, so these initiation rates (transition **e**) are set equal to 0. Cessation rates for new product users (transition **d**) are set equal to baseline smoking cessation rates and cessation rates for multiple product (any combination of current or former for both products) users (transitions **l**, **m**, **n**, **o**, and **p**) are also set equal to these smoking cessation rates.

The new product is assumed to have an excess relative risk that is equal to a proportion of the excess relative risk for cigarette smoking. Relative risks for current dual users are set equal to the maximum of the relative risks for the individual products. Former cigarette smokers who are current new product users are assumed to have lower ERR than current smokers, but higher ERR than they would have had had they quit tobacco products entirely. Table N details the scenario relative risk calculations and assumptions for all tobacco product use statuses.


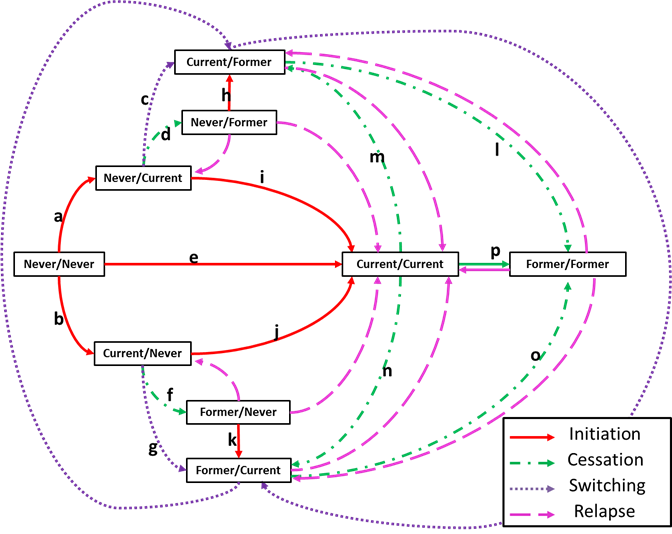


Figure F: Alternative two-product use states and transitions

**Figure Note**: Product Use #1/Product Use #2 represents users’ use of Product #1 (cigarettes) and Product #2 (hypothetical new product). Product use relapse is incorporated into the model but not included in the scenarios described in this manuscript.

Table M: Summary of two-product parameter inputs and implementation in model

| Parameter | Variable | Base Value | Range | Implementation in Model |
| --- | --- | --- | --- | --- |
| ***Health Risk of the New Product*** | | | | |
| Excess relative risk factor- a multiplicative factor that defines new product risks in terms of cigarette risks | $\alpha$ | 0.25 | [0.01,0.50] | All-cause mortality relative risk for a never smoker-current user of the new product is  ${RR}_{current new}=1+\alpha*({RR}_{current smoker}-1)$  All-cause mortality relative risk for a former smoker that switches to the new product is  ** |
| ***Impact on Current Smokers*** | | | | |
| Proportion of cigarette smokers who switch to new product use on an annual basis | *β* | 0.015 (A)* | [0,0.03] | $g\left( {2003}^{+} \right)=\beta$ |
|  |  | 0.010 (B)** |  |  |
| Proportion of cigarette smokers who transition to dual product use on an annual basis | *γ* | 0.015 (A)* | [0,0.03] | $j\left( {2003}^{+} \right)= \gamma$ |
|  |  | 0.020 (B)** |  |  |
| Proportion of switchers and dual users coming from smokers who would have otherwise quit smoking that year | *δ* | 0.25 | [0,0.5] | $f\left( {2003}^{+} \right)= f\left( 2000 \right)- \delta*\beta-\delta*\gamma$ |
| ***Impact on Never Smokers*** | | | | |
| New product initiation factor – represents the initiation rate for the new product among never smokers as a proportion of the cigarette smoking initiation rate | *ε* | 0.5 | [0.25,0.75] | $a\left( {2003}^{+} \right)= \varepsilon*b\left( 2000 \right)$ |
| Proportion of new product initiates who would have otherwise initiated cigarettes that year | *η* | 0.5 | [0.25,0.75] | $b\left( {2003}^{+} \right)=\left( 1-\varepsilon*\eta\right)*b({2000}^{+})$ |
| Proportion of new product users who switch to cigarette use on an annual basis | *θ* | 0.05 | [0,0.1] | $c\left( {2003}^{+} \right)=\theta$ |
| Proportion of new product users who transition to dual produce use on an annual basis | *ι* | 0.05 | [0,0.1] | $i\left( {2003}^{+} \right)= \iota$ |
| * Hypothetical scenario A in which transition rate to dual use and switching are equivalent  ** Hypothetical scenario B in which transition rate to dual use is greater than switching completely | | | | |

Table Note: $x\left( Y^{+} \right)$ represents the rate of transition with label $x$ from Figure S2.6. for year $Y$and subsequent years in the projection period.

Table N: Relative risk (RR) scenario assumption

| Tobacco Use Status* | Relative Risk Calculation | Interpretation |
| --- | --- | --- |
| NN | RR = 1 | Never/Never use represents the minimum risk state. |
| CN | RR for current smoker | CN is state for current smokers who do not use the new product. |
| FN | RR for former smoker | FN is state for former smokers who do not use the new product. |
| NC | RR = 1+ *α* *[ERR for current smoker] | Scenario assumes excess relative risk (ERR) for current user of the new product is a proportion of current smoker ERR defined by the excess relative risk factor *α* (from Table B.8). |
| CC | RR = maximum[RR for current smoker, RR for current new product use] | RR for dual use is the maximum of the individual RRs. |
| FC | RR = RR for former smoker +  *α* *[RR for current smoker – RR for former smoker] | FC is state for former smokers who are current users of the new product. Use of the new product is assumed to result in a lower RR than smoking but a higher RR than the FN population that quits tobacco entirely. We again use the excess relative risk factor *α* to define the RR in terms of the RR for current and former smokers. |
| NF | RR = 1 + *α* *[ERR for former smoker] | Scenario assumes ERR for former user of the new product is a proportion of former smoker ERR |
| CF | RR = Current-Smoker Risk | CF is state for current smokers who are former users of the new product. These individuals are assigned an RR equal to current smokers’ RR. |
| FF | RR = max(former smoker, former user) | FF is state for former smokers and former users of the new product. The individuals retain the RR from the highest risk behavior. |
| *First letter denotes cigarette use, and second letter denotes new product use. N=never, C=current, and F=former | | |

# S2.3 References

1. US Census Bureau (2008) 2008 National Population Projections.
2. National Center for Health Statistics (2013) National Health Interview Survey. http://www.cdc.gov/nchs/nhis.html.
3. US Census Bureau (2011) Statistical Abstract of the United States: 2012. Washington, DC.
4. National Center for Health Statistics (2013) NHIS Linked Mortality Files. http://www.cdc.gov/nchs/data_access/data_linkage/mortality/nhis_linkage.htm.
5. National Vital Statistics Report, National Center for Health Statistics (2004) Deaths: Final Data for 2002. Vol. 53, No. 5.
6. National Vital Statistics Report, National Center for Health Statistics (2006) Deaths: Final Data for 2003. Vol. 54, No. 13.
7. National Vital Statistics Report, National Center for Health Statistics (2007) Deaths: Final Data for 2004. Vol. 55, No. 19.
8. National Vital Statistics Report, National Center for Health Statistics (2008) Deaths: Final Data for 2005. Vol. 56, No. 10.
9. National Vital Statistics Report, National Center for Health Statistics (2009) Deaths: Final Data for 2006. Vol. 57, No. 14.
10. Lee RD, Carter LR (1992) Modeling and forecasting US mortality. Journal of the American Statistical Association 87: 659-671.
11. Anderson CM, Burns DM, Dodd KW, Feuer EJ (2012) Birth-cohort-specific estimates of smoking behaviors for the U.S. population. Risk Analysis 32 Suppl 1: S14-S24. doi: 10.1111/j.1539-6924.2011.01703.x.
12. Centers for Disease Control and Prevention (2011) Vital signs: current cigarette smoking among adults aged >/=18 years--United States, 2005-2010. MMWR Morb Mortal Wkly Rep 60: 1207-1212.
13. National Center for Health Statistics (2011) Health, United States, 2010. Hyattsville, MD.
14. Jha P, Ramasundarahettige C, Landsman V, Rostron B, Thun M et al. (2013) 21st-century hazards of smoking and benefits of cessation in the United States. N Engl J Med 368: 341-350. 10.1056/NEJMsa1211128.
15. Rostron B (2011) Smoking-attributable mortality in the United States. Epidemiology 22: 350-355. doi: 10.1097/EDE.0b013e3182126729.
16. van Meijgaard J, Fielding JE (2012) Estimating benefits of past, current, and future reductions in smoking rates using a comprehensive model with competing causes of death. Prev Chronic Dis 9: E122. E122.
17. Hyndman RJ (2013) demography: Forecasting mortality, fertility, migration and population data, version R package version 1.16 [computer program].
18. Preston, S., Heuveline, P., and Guillot, M. (2001) Demography: Measuring and Modeling Population Processes. Wiley.
19. Doll R, Peto R, Boreham J, Sutherland I (2004) Mortality in relation to smoking: 50 years' observations on male British doctors. BMJ 328: 1519. 10.1136/bmj.38142.554479.AE [doi];bmj.38142.554479.AE [pii].
20. Enstrom JE, Heath CW (1999) Smoking cessation and mortality trends among 118,000 Californians, 1960-1997. Epidemiology 10: 500-512. 00001648-199909000-00005 [pii].
21. Moolgavkar SH, Holford TR, Levy DT, Kong CY, Foy M et al. (2012) Impact of Reduced Tobacco Smoking on Lung Cancer Mortality in the United States During 1975–2000. Journal of the National Cancer Institute . 10.1093/jnci/djs136.
22. Feuer EJ, Levy DT, McCarthy WJ (2012) The impact of the reduction in tobacco smoking on U.S. lung cancer mortality, 1975-2000: An introduction to the problem. Risk Anal 32 Suppl 1: S6-S13. 10.1111/j.1539-6924.2011.01745.x [doi]. http://onlinelibrary.wiley.com/doi/10.1111/j.1539-6924.2011.01745.x/abstract;jsessionid 597781B73F78692FBB497F7E96DBDB53.f01t04
23. Freedman KS, Nelson NM, Feldman LL (2012) Smoking Initiation Among Young Adults in the United States and Canada, 1998-2010: A Systematic Review. Prev Chronic Dis . http://dx.doi.org/10.5888/pcd9.110037 .
24. Centers for Disease Control and Prevention (2014) Variance Estimation Guidance , NHIS 2006- 2013. http://www.cdc.gov/nchs/data/nhis/2006var.pdf
