# Supplementary material for: Modeling the Potential Effects of New Tobacco Products and Policies: A Dynamic Population Model for Multiple Product Use and Harm
Source: PLoS One. 2015 Mar 27;10(3):e0121008. doi: 10.1371/journal.pone.0121008 (PMC4376806; doi:10.1371/journal.pone.0121008)
Supplement: S3 Appendix — This appendix contains the results of model validation activities. Model projections for US population, cigarette smoking prevalence, and smoking attributable mortality are compared to published estimates from the US Census Bureau and US Centers for Disease Control and Prevention. (DOCX) [file pone.0121008.s003.docx]

**S3 Appendix: Model Diagnostics and Validation**

# S3.1. Method

To aid in model evaluation and validation, we present comparisons of results from our model and published estimates and projections. We first compare US population and mortality projections from our model with US Census Bureau projections for the period from 2000 to 2050. We also compare US adult cigarette smoking prevalence estimates from our model to estimates from the National Health Interview Survey (NHIS) for the period from 2000 to 2012. NHIS estimates are used by the Centers for Disease Control and Prevention (CDC) as estimates of smoking prevalence for the US population. We also present estimates of smoking-attributable deaths from our model in comparison with those from the CDC.

# S3.2. Results

## S3.2.1. Compare Population and Mortality Projections to Census

Fig. A presents US Census and model projections for the total US population from 2000 until 2050. The two sets of estimates are always very close, and the relative difference in a given year between the two estimates is always less than 0.8%. Fig. B contains projections of annual deaths in the US population from 2000 to 2050. The model estimates are initially lower than national estimates, but model and Census projections of deaths converge to a difference of less than 0.5% by 2050.

**
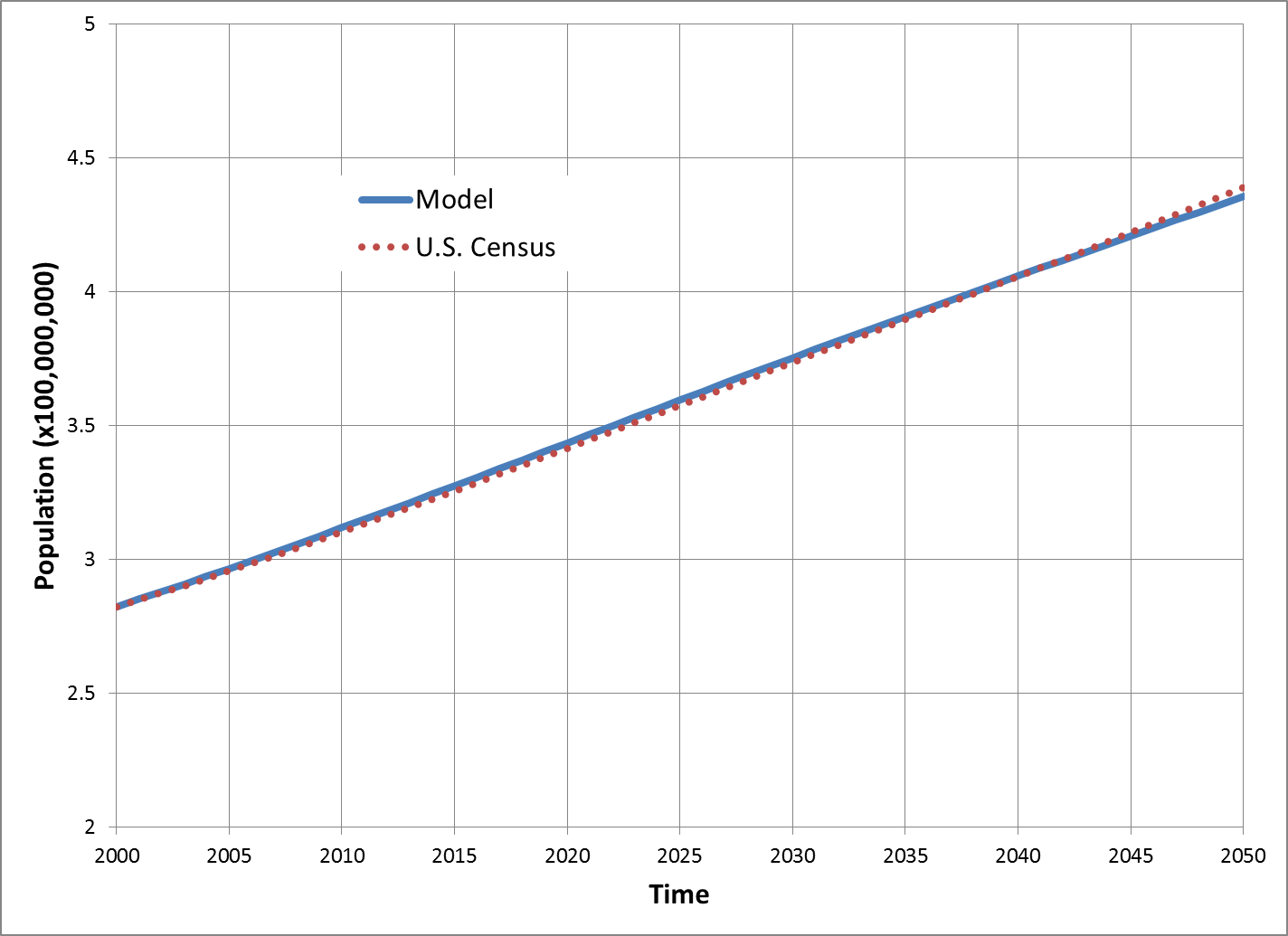
**

**Figure A: US total population projections**

Figure Note: Data Source: US Census Bureau [1]

**
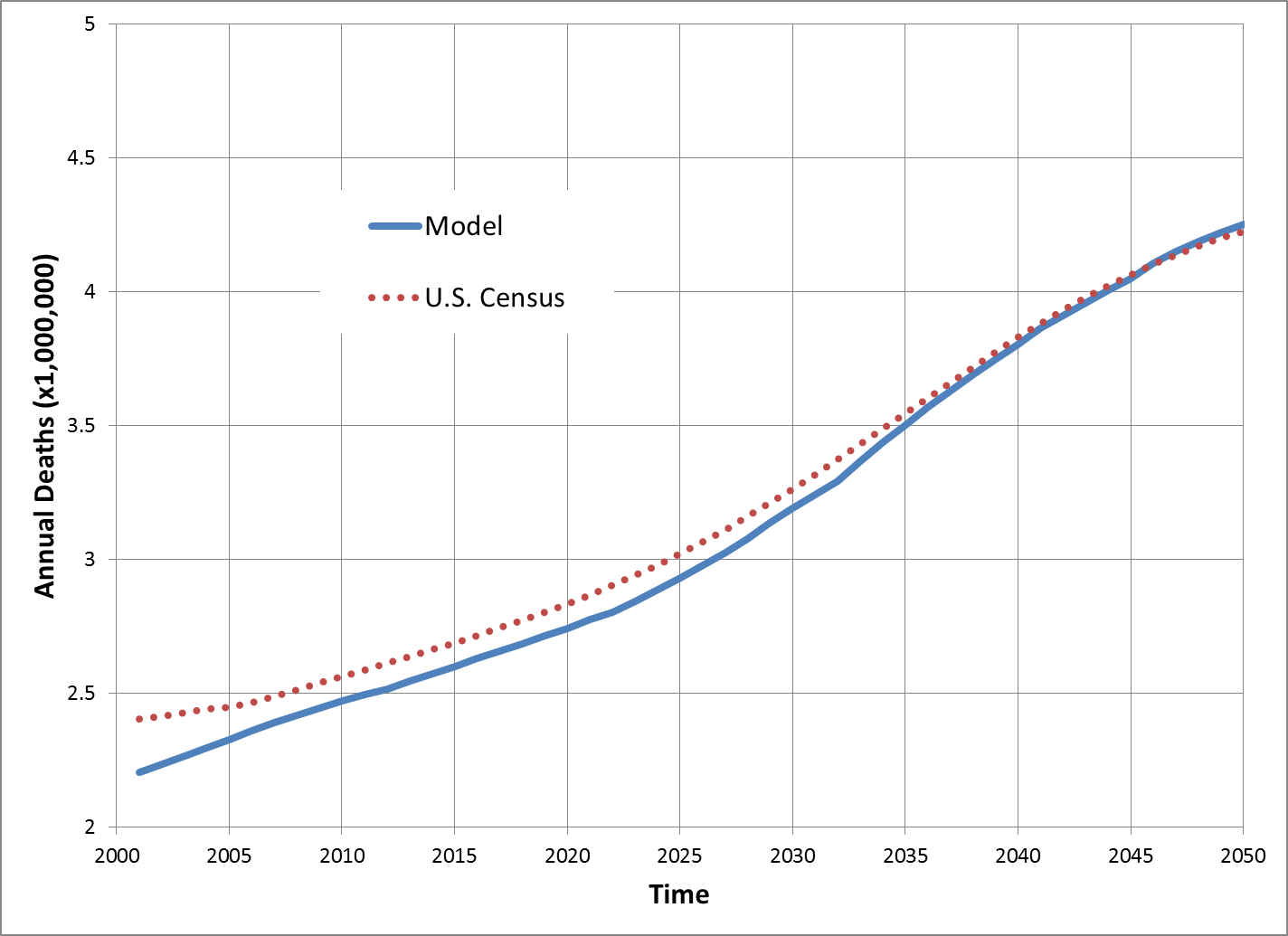
**

**Figure B: Projected US annual deaths**

**Figure Note**: Data Source: US Census Bureau [2]

## S3.2.2. Smoking Prevalence

Fig. C contains model projections from 2000-2050 and CDC observed estimates from 2000-2012 for smoking prevalence among US adults ages 18 years and older. Model estimates for 2000-2012 are comparable in magnitude to CDC estimates and show a similar decline over time. Fig. D presents estimates of US adult smoking prevalence by sex. Again, model projections and CDC estimates are comparable in magnitude and show similar decreases over time.


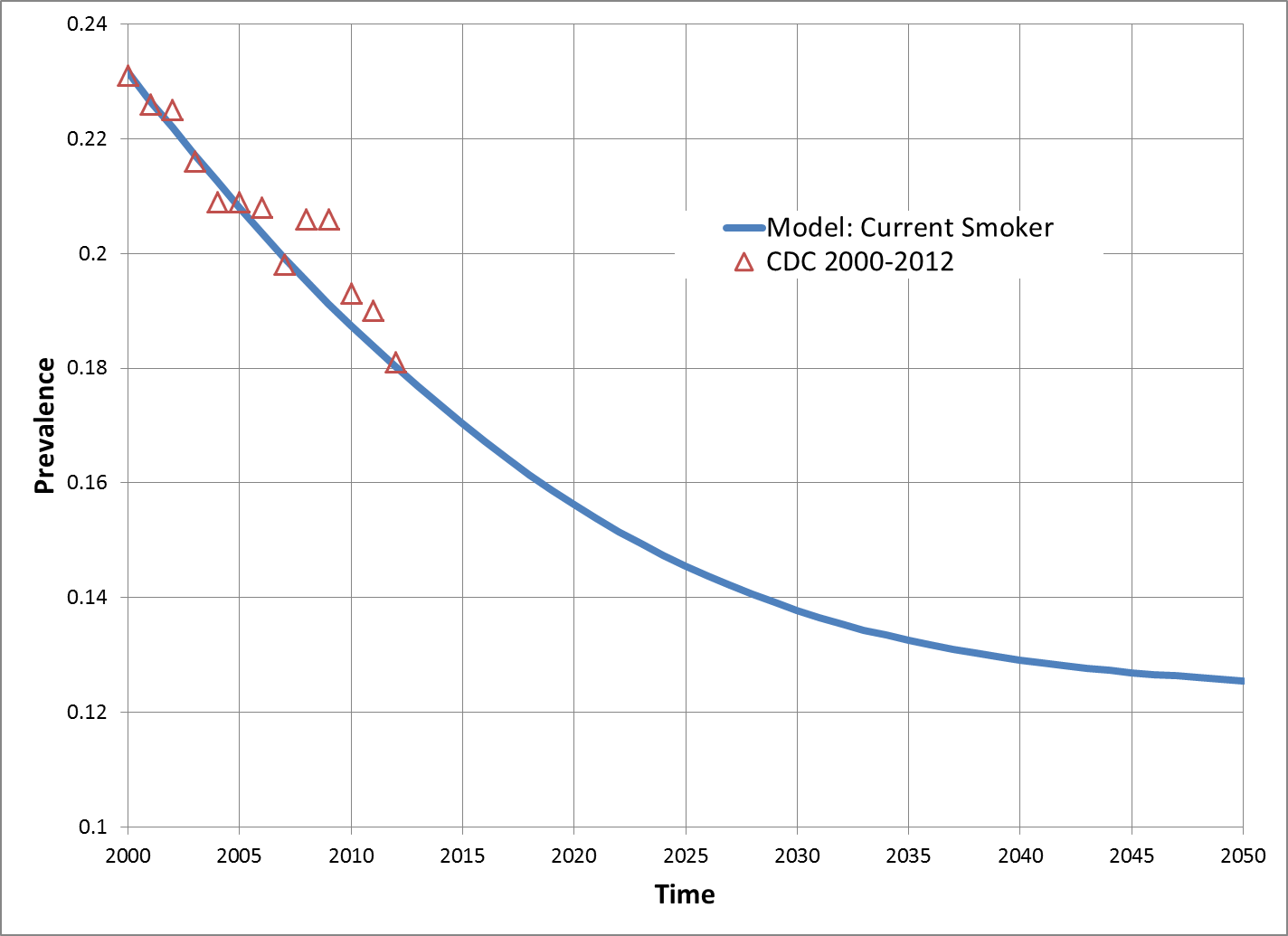


**Figure C: US adult smoking prevalence**

**Figure Note**: Data source: CDC [3]


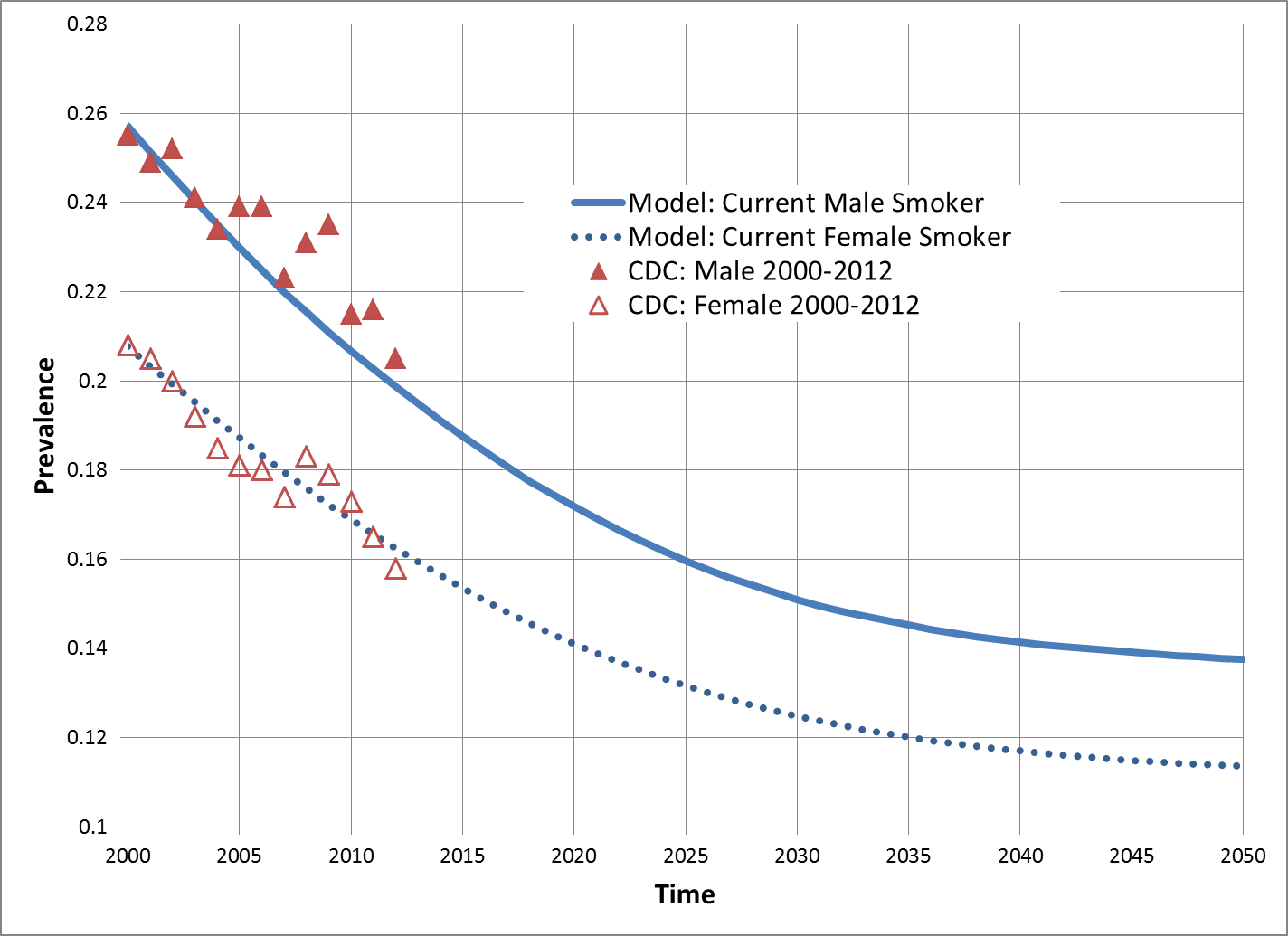


**Figure D: US adult smoking prevalence by sex**

**Figure Note**: Data source: CDC [3].

Table A shows estimated smoking prevalence by age group. Model projections are again similar to observed national estimates.

**Table A: US smoking prevalence by age**

|  | **Year** | **2000** | **2005** | **2010** |
| --- | --- | --- | --- | --- |
| **18-44** | Model | 0.270 | 0.245 | 0.227 |
|  | CDC | 0.267 | 0.241 | 0.215 |
| **45-64** | Model | 0.240 | 0.213 | 0.189 |
|  | CDC | 0.237 | 0.219 | 0.211 |
| **65-74** | Model | 0.129 | 0.125 | 0.116 |
|  | CDC |  | 0.111 | 0.130 |
| **75+** | Model | 0.058 | 0.052 | 0.039 |
|  | CDC |  | 0.058 | 0.051 |
| **65+** | Model | 0.095 | 0.088 | 0.079 |
|  | CDC | 0.096 |  |  |

**Table Note**: Data source: CDC [3]. In 2000 and 2001, CDC reported smoking prevalence for ages 65 years and older. In 2003, CDC changed their reporting format to report smoking prevalence for ages 65 to 74 years and 75 years and older.

## S3.2.3. Smoking-Attributable Mortality

Our model can be used to estimate smoking-attributable mortality (SAM) and the smoking-attributable fraction of deaths in the US population. Most estimates of current smoking-attributable mortality for the US are generally in the range of 400,000 to 500,000 deaths per year [4], [5], [6], [7]. Table B lists smoking-attributable mortality estimates from the model and from the CDC [5] for the years 2000 to 2004 and 2005 to 2009.

Estimates from our model and the CDC are generally comparable. The CDC updated the data and methodology that it uses to estimate smoking-attributable mortality for 2005 to 2009, thus explaining the increase in smoking-attributable deaths from the earlier period in its estimates. The CDC previously used relative risks obtained from American Cancer Society Cancer Prevention Study II (CPS-II) data in its estimation of smoking-attributable mortality for the US [4]. CPS-II was a large longitudinal cohort study with over one million participants that was initiated in 1982. The CDC used mortality follow-up from the CPS-II from 1982 to 1988 in its Smoking-Attributable Mortality, Morbidity, and Economic Costs (SAMMEC) methodology [8]. Research has shown that relative risks for smoking have changed since this time and that risks for women have come to be similar to those for men [9]. The CDC updated its data and methods for estimating smoking-attributable mortality in the 2014 Surgeon General’s Report on the health effects of smoking in producing its estimates of smoking-attributable mortality for the US from 2005 to 2009 [5]. The estimates are obtained from relative risks from mortality follow-up for a pooled analysis of several more recent cohort studies, and the estimates are calculated for more specific age groups for some causes of death. Our method and the CDC method estimate smoking-attributable mortality in somewhat different ways, given that we estimate smoking-attributable mortality from all-cause mortality whereas the CDC estimates smoking-attributable mortality for a set of smoking-related causes, but the two sets of estimates are generally consistent. We use relative risks obtained from NHIS-LMF data, given that this data source provides recent, nationally representative estimates from data collected and made publicly available by the CDC’s National Center for Health Statistics, the federal government’s principal health statistics agency. Our estimates for US smoking-attributable mortality from 2000-2009 are consistent with published estimates for this period calculated with NHIS-LMF relative risks using the SAMMEC methodology previously used by CDC [6].

Table B also includes estimated smoking-attributable fractions of deaths for the US, which is calculated by dividing the number of smoking-attributable deaths by the total numbers for an age group in a time period. Model estimates are again comparable to CDC estimates and the smoking-attributable fractions of deaths reported by researchers using a variety of estimation methods (see Table 4 in Fenelon and Preston [10] for a collection of different approaches and corresponding estimates of smoking-attributable fraction of deaths).

Table B: Smoking-attributable mortality and smoking-attributable fraction of deaths:
ages 35 years and older

|  | Total Smoking-Attributable Mortality | | Average Annual Smoking-Attributable Mortality^+^ | | Average Annual Smoking-Attributable Fraction of Deaths | |
| --- | --- | --- | --- | --- | --- | --- |
| Year | Model | CDC*^,^** | Model | CDC | Model | CDC^++,+++^ |
| 2000-2004 | 2,080,000 | 1,960,000 | 416,000 | 392,000 | 0.18 | 0.17 |
| 2005-2009 | 1,950,000 | 2,190,000 | 390,000 | 438,000 | 0.16 | 0.19 |
| *Data Source: US Department of Health and Human Services [5].  **Model projections of smoking-attributable mortality do not include deaths from secondhand smoke exposure and residential fires and perinatal deaths. For purposes of comparison, the CDC estimates reported in the table do not include these categories of deaths as well.  ^+^Average Annual Smoking-Attributable Mortality = (Total Smoking-Attributable Mortality)/5 years  ^++^Average Annual Smoking-Attributable Fraction of Deaths =  (Smoking-Attributable Mortality in 5 year period)/(Total Number of Deaths in 5 year period)  ^+++^Data source for total number of deaths: CDC [11]. | | | | | | |

# S3.3 Conclusion

Model projections for the US are similar to national estimates and projections for population size, deaths, cigarette smoking prevalence, and smoking-attributable mortality.

# S3.4 References

1. US Census Bureau 2008a (2008) Projected Population by Single Year of Age, Sex, Race, and Hispanic Origin for the United States: July 1, 2000 to July 1, 2050.
2. US Census Bureau 2008b (2008) Projected Deaths by Sex, Race, and Hispanic Origin for the United States: July 1, 2000 to June 30, 2050.
3. Centers for Disease Control and Prevention (2014) NCHS Vital Health Statistics Series 10. Data From the National Health Interview Survey. Numbers 215, 218, 222, 225, 228, 232, 235, 240, 242, 249, 252, and 256.
4. Centers for Disease Control and Prevention (2008) Smoking-attributable mortality, years of potential life lost, and productivity losses-- United States, 2000-2004. Morbidity and Mortality Weekly Report Vol.57, No. 45: 1226-1228.
5. Centers for Disease Control and Prevention (2014) The Health Consequences of Smoking: 50 Years of Progress. A Report of the Surgeon General.
6. Rostron B (2008) Smoking-Attributable Mortality by Cause in the United States: Revising the CDC's Data and Estimates. Nicotine and Tobacco Research 15: 238-246.
7. National Research Council (2011) International Differences in Mortality at Older Ages: Dimensions and Sources. National Academies Press.
8. Centers for Disease Control and Prevention (2014) Smoking-Attributable Mortality, Morbidity, and Economic Costs (SAMMEC).
9. Thun M, Carter B, Feskanich D, Freedman N, Prentice R et al. (2013) 50-Year Trends in Smoking-Related Mortality in the United States. New England Journal of Medicine 368: 351-364.
10. Fenelon A, Preston S (2012) Estimating Smoking-Attributable Mortality in the United States. Demography Vol. 49, No. 3: 797-818.
11. Centers for Disease Control and Prevention, CDC Wonder Online Databases (2013) Detailed Mortality: About Underlying Cause of Death, 1999-2010.
